# Nomimicins B–D, new tetronate-class polyketides from a marine-derived actinomycete of the genus *Actinomadura*

**DOI:** 10.3762/bjoc.17.141

**Published:** 2021-08-27

**Authors:** Zhiwei Zhang, Tao Zhou, Taehui Yang, Keisuke Fukaya, Enjuro Harunari, Shun Saito, Katsuhisa Yamada, Chiaki Imada, Daisuke Urabe, Yasuhiro Igarashi

**Affiliations:** 1 Biotechnology Research Center and Department of Biotechnology, Toyama Prefectural University, 5180 Kurokawa, Imizu, Toyama 939-0398, Japan; 2 Graduate School of Marine Science and Technology, Tokyo University of Marine Science and Technology, 4-5-7, Konan, Minato-ku, Tokyo 108-8477, Japan; 3 DHC Corporation, 2-7-1 Minami-Azabu, Minato-ku, Tokyo 106-8571, Japan

**Keywords:** *Actinomadura*, nomimicin, polyketide, spirotetronate

## Abstract

Three new tetronate-class polyketides, nomimicins B, C, and D, along with nomimicin, hereafter named nomimicin A, were isolated from the culture extract of *Actinomadura* sp*.* AKA43 collected from floating particles in the deep-sea water of Sagami Bay, Japan. The structures of nomimicins B, C, and D were elucidated through the interpretation of NMR and MS analytical data, and the absolute configuration was determined by combination of NOESY/ROESY and ECD analyses. Nomimicins B, C, and D showed antimicrobial activity against Gram-positive bacteria, *Kocuria rhizophila* and *Bacillus subtilis*, with MIC values in the range of 6.5 to 12.5 μg/mL*.* Nomimicins B and C also displayed cytotoxicity against P388 murine leukemia cells with IC_50_ values of 33 and 89 μM, respectively.

## Introduction

Actinomycetes are a valuable source of bioactive compounds, accounting for approximately two thirds of all known antibiotics, and more than 70% of them are produced by the genus *Streptomyces* [1]. However, intensive and concentrated screening activities on soil actinomycetes resulted in the repeated isolation of known compounds [2], which consequently prompted the exploration of untouched niches, such as extreme environments [3]. The marine environment is now attracting attention as a promising source for actinomycetes potentially producing new secondary metabolites [4–5]. Indeed, a large number of pharmaceutically worthy new compounds has been found from marine actinomycetes in various niches including sediments, sea water, marine plants, and marine invertebrates [6–8]. In the past 20 years, our laboratory studied the actinomycetes collected from the deep sea water (DSW) of the Sea of Japan and discovered diverse classes of bioactive compounds, such as lydicamycin congeners, γ-butyrolactones, and nyuzenamides [9–11], whereas DSW in other sea areas had not been studied, although a total of 15 DSW pumping stations are operated in Japan. We recently carried out metagenomic analysis of DSW using DGGE and pyrosequencing techniques and revealed that the bacterial community structure in DSW was varied depending on the collection sites [12]. Furthermore, we found that the DSW of Sagami Bay (Pacific Ocean side of Honshu Island, Japan) contained more unknown actinomycete species than other sea areas, which eventually led to the discovery of akazamicin, a new cytotoxic aromatic polyketide from *Nonomuraea* [13] and akazaoxime, an antibacterial oxime derivative from *Micromonospora* [14]. Along the lines of these previous studies, metabolite analysis of actinomycetes from the DSW of Sagami Bay was further conducted, and three new tetronate-class polyketides, nomimicins B (**1**), C (**2**), and D (**3**) were found from a rare actinomycete of the genus *Actinomadura*. We herein describe the isolation, structure determination, and biological activities of **1**‒**3**.

## Results and Discussion

**Figure 1:**
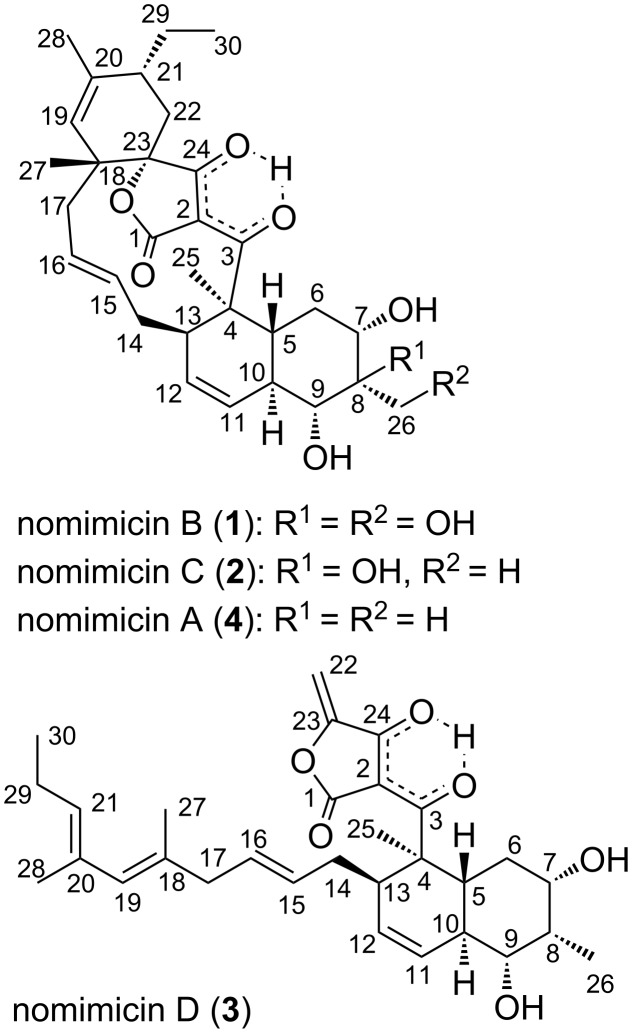
Structures of nomimicins A–D (**4** and **1**–**3**).

The producing strain *Actinomadura* sp. AKA43 was isolated from DSW collected at a depth of −800 m in Sagami Bay, Japan. Strain AKA43 was cultured in A16 medium, and the whole culture broth was extracted with 1-butanol. The extract was subjected to silica gel and ODS column chromatography, and the final purification was achieved by reversed-phase HPLC to yield two new spirotetronate polyketides, nomimicins B (**1**) and C (**2**), along with a known compound, nomimicin A (**4**) [15]. From the extract of the fermentation broth cultured in A11M medium, an additional new tetronate polyketide, nomimicin D (**3**), was isolated (Figure 1).

**Figure 2:**
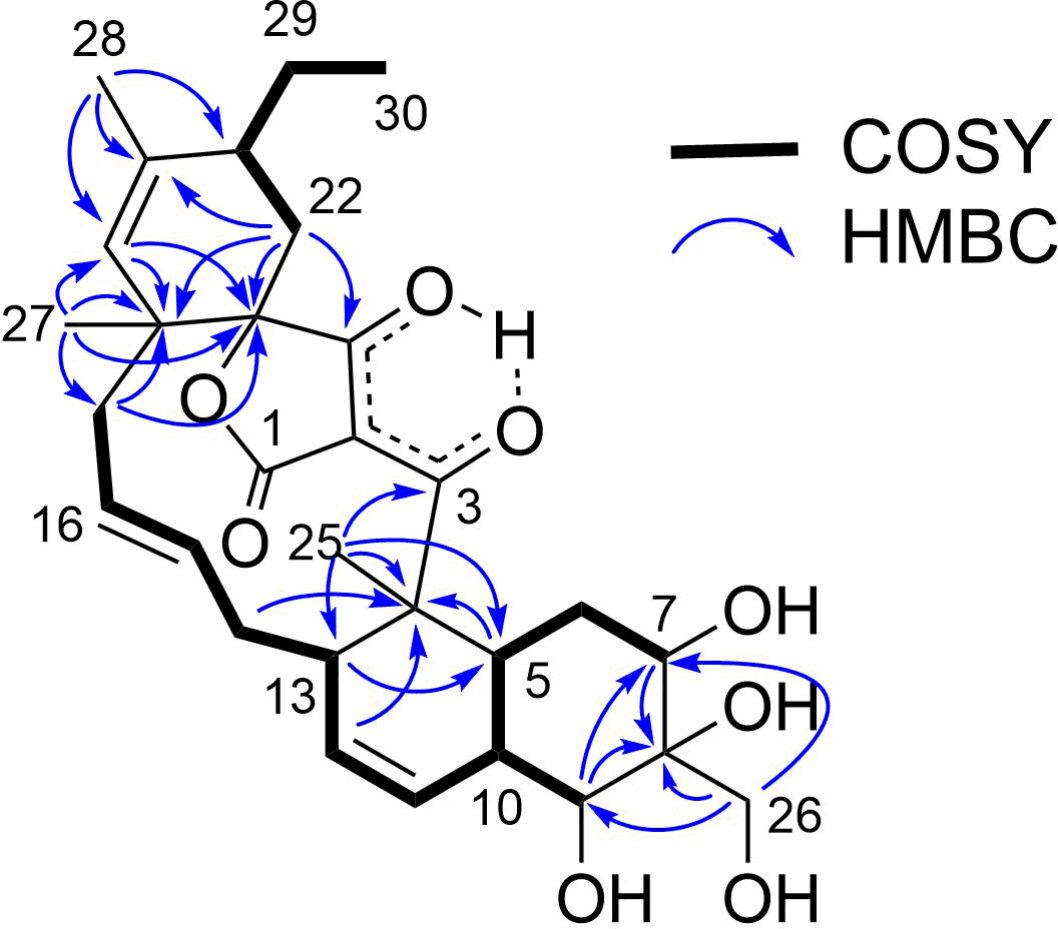
COSY and key HMBC correlations for **1**.

**Figure 3:**
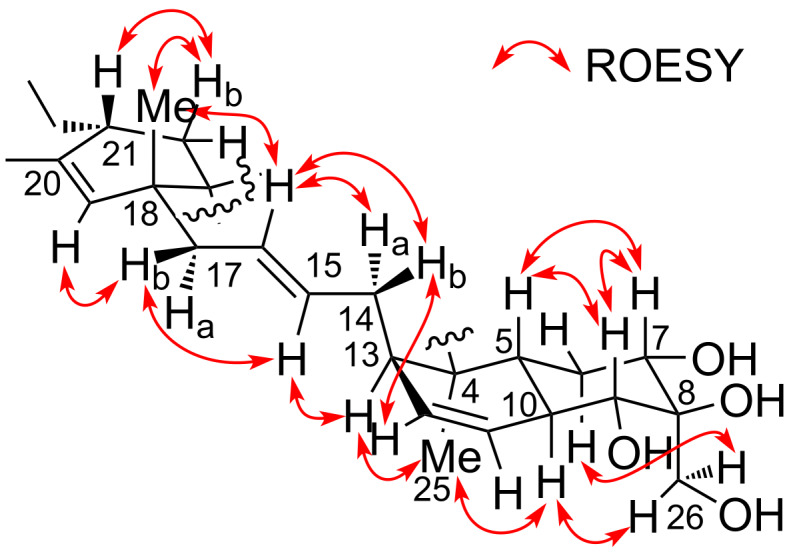
Relative configuration of **1** determined by ROESY analysis.

**Figure 4:**
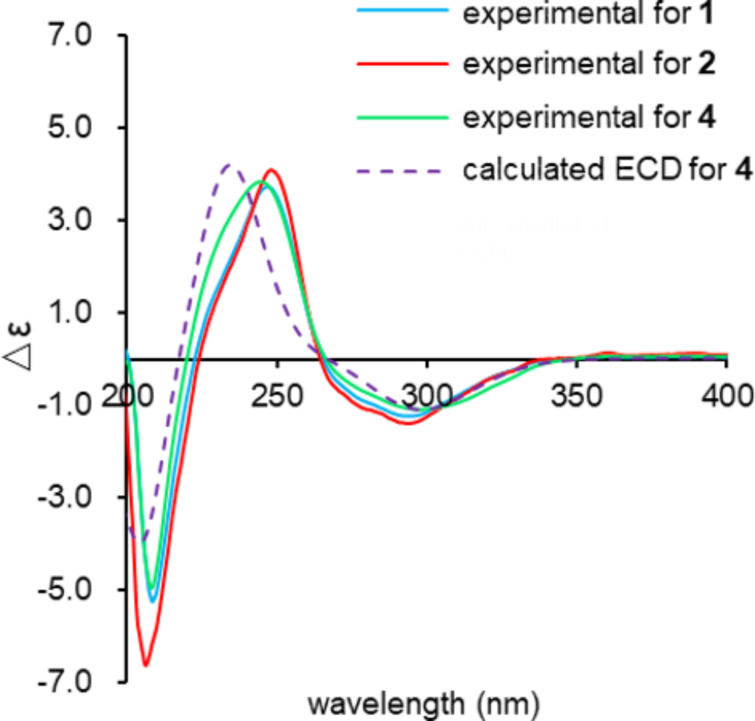
Experimental ECD spectra of **1**, **2**, and **4** and calculated ECD spectrum of **4**.

**Figure 5:**
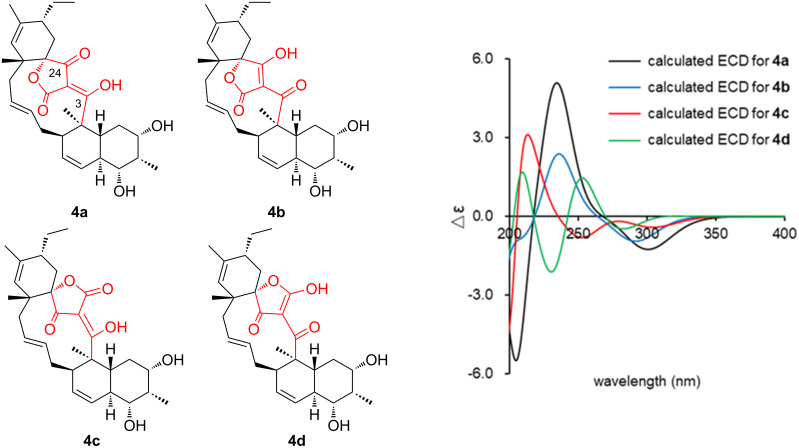
Four tautomers **4a**–**4d** of the tetronic acid moiety of **4** and calculated ECD spectra.

Nomimicin B (**1**) was obtained as a colorless amorphous solid. The molecular formula was determined to be C_30_H_40_O_8_, based on the HRESITOFMS analysis ([M + Na]^+^ *m*/*z* 551.2612, Δ −0.3 mmu). Structural analogy between **1** and **4** was suggested by the global similarity of the UV and NMR spectra between the compounds. In the ^13^C NMR spectrum, four nonprotonated carbons assignable to the tetronic acid moiety were detected at δ_C_ 108.2, 170.4, 200.7, and 204.5. In addition, ^13^C NMR and HSQC analyses revealed the presence of six sp^2^ carbon atoms (five are proton-bearing), two quaternary sp^3^ carbon atoms, two oxygen-bearing nonprotonated carbon atoms, six sp^3^ methine groups (two are oxygenated), six sp^3^ methylene moieties, and four methyl groups (Table 1). COSY analysis clarified two carbon chains (Figure 2). The first one, spanning from H7 to H17, contained an oxymethine (H9) branching at C10, a *cis*-configured double bond between C11 and C12 (^3^*J*_H11,H12_ = 10.0 Hz), and a *trans*-double bond between C15 and C16 (^3^*J*_H15,H16_ = 14.8 Hz). Placement of the oxygenated carbon atom C8 between C7 and C9 and the attachment of the oxygenated methylene carbon atom C26 to C8 were deduced from the HMBC correlations from H7 and H9 to C8 and C26 and from H26 to C7, C8, and C9, thereby establishing a highly oxygenated cyclohexane ring. This six-membered ring was fused with another six-membered ring to give a dehydrodecalin core by the correlations from a singlet methyl hydrogen atom H25 to C4, C5, and C13. A series of HMBC correlations from two methyl singlets H27 and H28 elucidated the carbon connectivity of C23–C18–C19–C20–C21 (Figure 2), which was then coupled with the other COSY-defined fragment, C22/C21/C29/C30, by the correlations from H22 to C18 and C23, yielding a cyclohexene ring. This ring was joined together with the dehydrodecalin moiety at the quaternary carbon atom C18 by HMBC correlations from H17 to C18, C19, and C23. As for the remaining four sp^2^ carbon atoms, C1, C2, C3, and C24, although only limited HMBC correlations H22/C24 and H25/C3 were available, a spirotetronate structure was assembled. This was in consideration of the high similarity of the ^13^C NMR chemical shifts of these carbon atoms to those for the corresponding carbon atoms in **4** as well as due to the closeness of the UV spectral pattern between **1** and **4**. The remaining four protons were finally assigned to hydroxy groups at C7, C8, C9, and C26 to complete the planar structure of **1**. While **4** has an axial methyl group at C8 and equatorial hydroxy groups at C7 and C9, **1** has two additional hydroxy groups at C8 and C26. The axial orientation of the C26 hydroxymethyl group was supported by the ROESY correlations H26/H6ax and H26/H10 (Figure 3). The relative configuration of the remaining part was determined to be identical with **4** on the basis of ROESY correlations (Table S1, Supporting Information File 1) and ^3^*J*_HH_ coupling constants [15]. The absolute configuration of **1** was deduced to be the same as **4** in consideration of the overall similarity of the electronic circular dichroism (ECD) spectra of **1** and **4** (Figure 4). This proposition was evidenced by the density functional theory (DFT) calculation of the ECD spectrum for **4**, for which the absolute configuration was established by the modified Mosher’s method in our previous work [15]. Since the acyltetronic acid exists as a mixture of keto–enol tautomers, the calculation was carried out using the four possible canonical structures of **4** (**4a**–**d** in Figure 5). The calculated ECD spectra of **4a**–**d** and the one of **4**, which includes all contributions from each tautomer according to the energy distribution, are shown in Figure 5 and Figure 4, respectively. The experimental ECD spectrum of **4**, with positive and negative Cotton effects at 244 and 298 nm, matched well with the calculated one of **4** (Figure 4). Noteworthy is that **4a** and **4b** (Tables S4–S7, Supporting Information File 1) have a lower free energy than **4c** and **4d**, and the calculated spectra for **4a** and **4b** are similar to the experimental one, indicating that **4a** and **4b** are the dominant tautomers in solution. This is the first validation to state that the keto carbonyl unit in the five-membered ring (C24 in Figure 5) and the acyl ketone connecting at C2 (C3 in Figure 5) are preferably enolized to form stable isomeric structures in spirotetronic acids.

**Table 1:** ^1^H and ^13^C NMR data for nomimicins B (**1**) and C (**2**).

	nomimicin B (**1**)			nomimicin C (**2**)		
atom no.	δ_C_^a^	δ_H_, mult (*J* in Hz)^b^	HMBC^b,c^	δ_C_^a^	δ_H_, mult (*J* in Hz)^a^	HMBC^b,c^

1	170.4			169.9		
2	108.2			108.4		
3	200.8			201.0		
4	51.0			51.0		
5	36.6	1.66^d^	4, 7, 9, 25	36.4	1.68^d^	4, 6, 7, 9, 10, 25
6ax	34.3	1.34, ddd (12.0, 12.0, 12.0)	5, 7, 10	34.1	1.20, ddd (11.9, 11.9, 11.9)	5, 7, 8, 10
6eq		2.41, brd (12.0)	7		2.35^d^	5, 7, 8, 10
7	77.4	3.74, dd (12.0, 4.3)	5, 6, 8, 26	76.8	3.62, dd (11.8, 4.2)	5, 6, 8, 26
8	77.4			79.1		
9	80.5	3.21, d (11.2)	5, 7, 8, 10, 11, 26	79.8	3.11, d (11.0)	5, 8, 10, 11, 26
10	41.8	2.02^d^		41.7	1.85^d^	
11	124.5	5.85, d (10.0)	5, 9, 13	124.8	5.84, d (10.0)	5, 9, 10, 13
12	132.0	5.61, ddd (10.0, 5.3, 2.6)	4, 10, 13	131.6	5.60, ddd (10.0, 5.1, 2.5)	4, 10, 13
13	39.4	2.81, m	4, 11, 12, 14, 25	39.4	2.79, m	4, 5, 11, 12, 14, 15, 25
14a	37.6	1.80^d^	4, 13, 15, 16	37.6	1.80^d^	4, 13, 15, 16
14b		1.98^d^	16		1.98^d^	15, 16
15	137.5	5.49, dd (14.7, 11.5)		137.6	5.48, dd (14.5, 11.9)	
16	124.8	5.12, dd (14.8, 11.3)		124.8	5.12, dd (14.8, 11.6)	
17a	44.1	1.95^d^	15, 16	44.0	1.95^d^	15, 16, 18, 19, 23
17b		2.32^d^	15, 16, 27		2.32^d^	15, 16, 19, 27
18	40.8			40.7		
19	130.8	5.00, s	17, 18, 23, 28	130.7	5.01, s	17, 18, 21, 23, 28
20	135.1			135.1		
21	40.5	2.00^d^		40.5	2.01^d^	
22a	30.7	1.78^d^	18, 20, 21, 23, 24, 29	30.7	1.79^d^	18, 21, 23, 24, 29
22b		2.34^d^	18, 21, 29		2.34^d^	21, 29
23	87.9			88.0		
24	204.6			204.7		
25	16.6	1.60, s	3, 4, 5, 13	16.6	1.59, s	3, 4, 5, 13
26	62.9	3.99, s	7, 8, 9	13.9	1.15, s	7, 8, 9
27	24.4	1.24, s	17, 18, 19, 23	24.3	1.25, s	17, 18, 19, 23
28	22.5	1.75, s	19, 20, 21	22.4	1.75, s	19, 20, 21
29a	26.4	1.58^d^	22, 30	26.4	1.62^d^	21, 22, 30
29b		1.72^d^	30		1.75^d^	20, 22, 30
30	13.2	0.93, t (7.4)	21, 29	13.1	0.93, t (7.4)	21, 29

^a^Recorded at 125 MHz. ^b^Recorded at 500 MHz. ^c^From proton to indicated carbon atom(s). ^d^Overlapping signals.

The molecular formula of nomimicin C (**2**) was determined as C_30_H_40_O_7_ on the basis of HRESITOFMS data ([M + Na]^+^ *m*/*z* 535.2665, Δ −0.1 mmu), indicating that one oxygen atom (16 amu) was less compared to **1**. This was consistent with the NMR spectra of **2** in which the resonances for the hydroxylated methylene moiety H26/C26 (δ_H_ 3.99, δ_C_ 62.9) disappeared, and those for a shielded methyl group (δ_H_ 1.15, δ_C_ 13.9) appeared instead, implying that the C26 hydroxymethyl group in **1** was replaced by a methyl group in **2**. HMBC correlations from H26 to C7, C8, and C9 and ROESY correlations between H26 and H10 supported the presence of a methyl group at C8 in an axial orientation (Figure S36, Supporting Information File 1). The remaining part of **2** was constructed based on COSY and HMBC analyses, and the relative configuration was established by NOESY/ROESY analyses (Table S2, Supporting Information File 1). The close similarity of ECD spectra between **1** and **2** was also indicative of the same absolute configuration of **2** and **4** (Figure 4).

**Figure 6:**
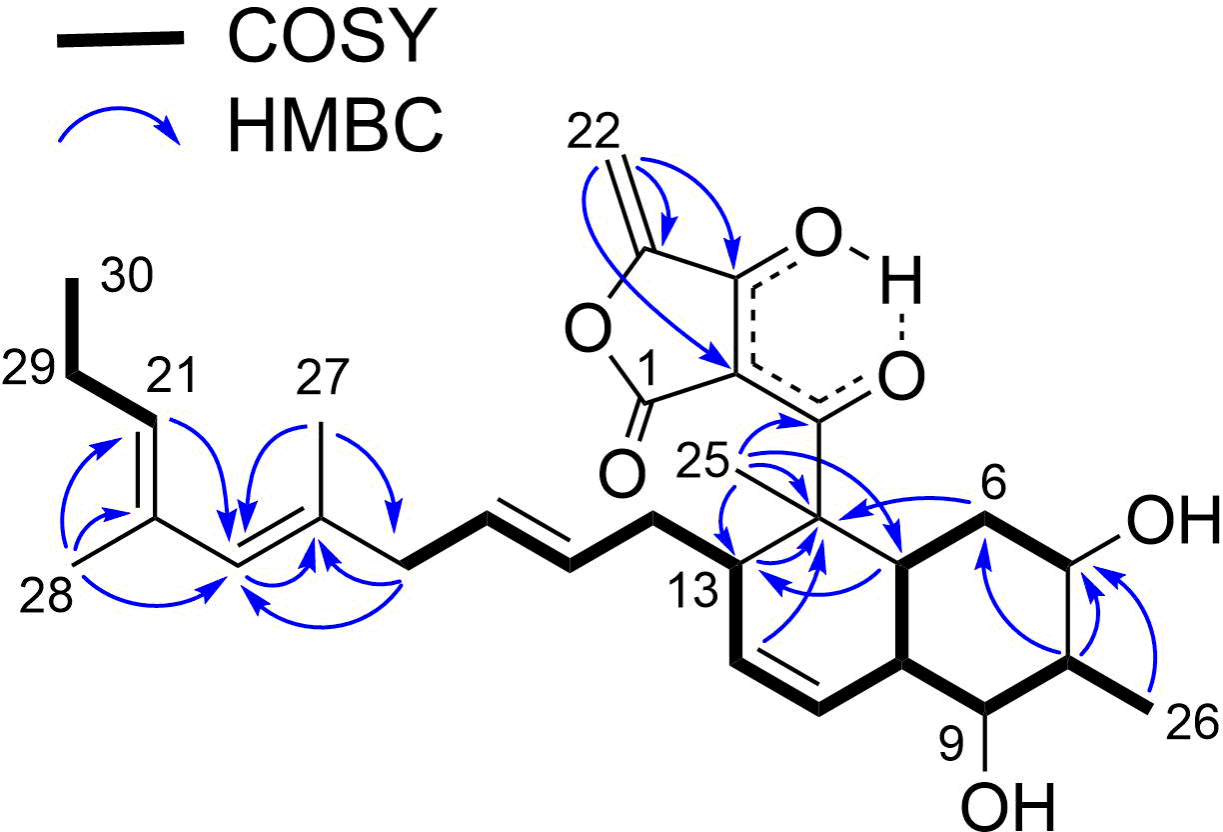
COSY and key HMBC correlations for **3**.

The molecular formula of nomimicin D (**3**) was determined to be C_30_H_42_O_6_ through the HRESITOFMS analysis, which gave a sodium adduct ion [M + Na]^+^ at *m*/*z* 519.2717 (Δ 0.0 mmu). Analysis of ^1^H NMR, ^13^C NMR, and HSQC spectra revealed the presence of four oxygenated sp^2^ carbon atoms, three nonprotonated sp^2^ carbon atoms, six sp^2^ methine groups, one sp^2^ methylene unit, one quaternary carbon atom, six sp^3^ methine groups (two are oxygenated), four sp^3^ methylene units, and five sp^3^ methyl groups (Table 2). A sequence of COSY correlations from the doublet methyl proton H26 to an oxymethine proton H7 via an oxymethine proton H9, together with HMBC correlations from H26 and H8 to C7 and H8 to C6, gave an oxygenated cyclohexane ring with a methyl substitution (Figure 6). COSY correlations were extended from H10 to a methylene proton H17, providing a carbon chain containing double bonds at C11/C12 and C15/C16. HMBC correlations from a singlet methyl hydrogen atom H25 to C4, C5, and C13 indicated closure of another cyclohexane ring, and thus a dehydrodecalin core with a side chain at C13 was established. The chain was further extended from H17 to the terminal methyl proton H30 by COSY correlations for H21/H29/H30 and a series of HMBC correlations from two allylic methyl hydrogen atoms H27 and H28 to the carbon atoms within a three-bond length (Figure 6). The remaining five nonprotonated carbon atoms, C1 (δ_C_ 174.5), C2 (δ_C_ 99.5), C3 (δ_C_ 203.2), C23 (δ_C_ 155.7), and C24 (δ_C_ 180.7) and the *exo*-methylene group (H22: δ_H_ 4.66/5.00; C22: δ_C_ 88.7) were assigned to the tetronic acid moiety based on the following considerations. First, the ^13^C chemical shift values of these six carbon atoms were closely similar to those for the tetronic acid bearing an *exo*-methylene substituent in known natural products [16–18]. Secondly, the UV spectrum of **3**, showing absorption maxima at 243 and 302 nm, was matched well with that for ecteinamycin, which possesses the *exo*-methylene-substituted tetronic acid moiety [19]. This assignment was supported by correlations from H22 to C23, C24, and C2 and from H25 to C3, though not all the carbon–carbon connectivities were proven by HMBC analysis.

**Table 2:** ^1^H and ^13^C NMR data for nomimicin D (**3**).

	nomimicin D (**3**)		
no.	δ_C_^a^	δ_H_, mult (*J* in Hz)^b^	HMBC^b,c^

1	174.5		
2	99.5		
3	203.2		
4	52.8		
5	36.5	1.72^d^	4, 7, 9, 11, 25
6ax	30.8	1.14, ddd (11.7, 11.7, 11.7)	4, 5, 7, 8, 10
6eq		1.80, brd (11.7)	7
7	72.3	3.83, ddd (11.6, 4.5, 4.5)	26
8	43.4	2.32, m	7, 9, 10, 26
9	75.5	3.40, dd (10.8, 4.7)	5, 10, 11, 26
10	38.9	1.94^d^	9, 11
11	125.8	5.85, d (10.2)	5, 9, 10, 12, 13
12	131.5	5.72, ddd (10.2, 4.8, 2.5)	4, 13, 14
13	41.5	3.32^d^	4, 5, 14
14a	38.7	1.75^d^	12, 13, 15, 16
14b		2.00^d^	12, 13, 15, 16
15	131.8	5.40, dt (15.0, 7.2)	14, 16, 17
16	130.7	5.26, dt (15.2, 7.0)	14, 15, 17
17	44.9	2.63, d (6.9)	15, 16, 18, 19, 27
18	135.6		
19	130.5	5.59, s	17, 21, 27, 28
20	133.5		
21	131.9	5.20, t (7.3)	19, 29, 26
22a	88.7	4.66, d (1.5)	2, 23, 24
22b		5.00, d (1.5)	23, 24
23	155.7		
24	180.7		
25	16.0	1.38, s	3, 4, 5, 13
26	6.1	0.92, d (6.9)	7, 8, 9
27	18.2	1.69, s	17, 18, 19
28	17.2	1.67, s	19, 20, 21
29	22.5	2.08, q (7.5)	20, 21, 30
30	14.8	0.98, t (7.5)	21, 29

^a^Recorded at 125 MHz. ^b^Recorded at 500 MHz. ^c^From proton to indicated carbon atom(s). ^d^Overlapping signals.

**Figure 7:**
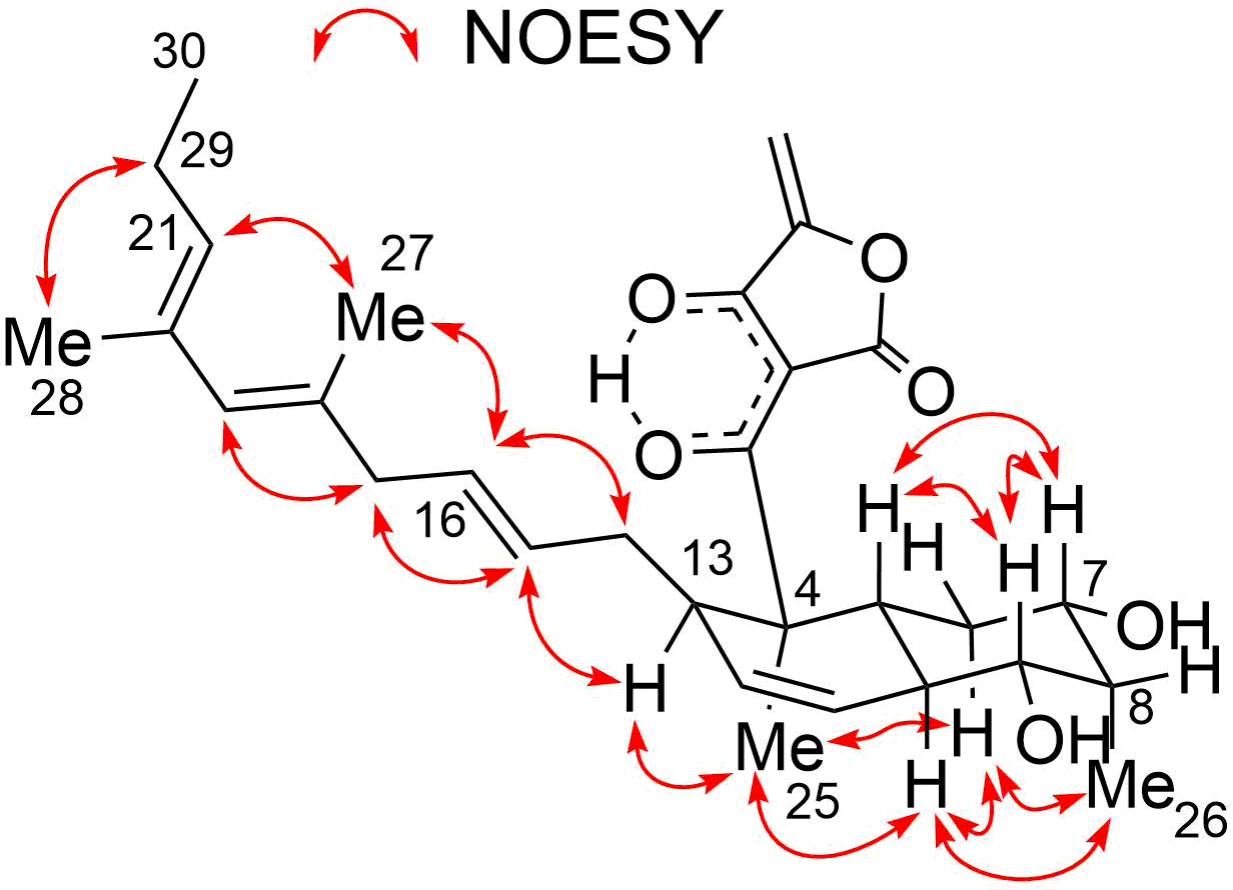
Relative configuration of **3** determined by NOESY analysis.

The relative configuration of **3** was elucidated by analyzing the NOESY spectrum (Table S3, Supporting Information File 1). Correlations for H5/H7, H5/H9, and H7/H9 and large scalar couplings (^3^*J*_HH_ > 10 Hz) for H5/H6ax, H6ax/H7, and H5/H9 established the *trans*-ring fusion of the dehydrodecalin moiety. The axial orientation of the methyl group at C8 was evidenced by NOESY correlations H26/H6ax and H26/H10. Correlations of H25/H10 and H25/H13 placed the H25 methyl proton and H13 on the same side of the dehydrodecalin ring at the H9 axial proton. The geometries of the double bonds at C15/C16, C18/C19, and C20/C21 were assigned as *E*, based on the NOESY correlations H13/H15, H14/H16, H15/H17, H17/H19, and H28/H29 (Figure 7). The s-*cis* configuration of the diene moiety was indicated by a NOESY correlation between H21 and H27. The absolute configuration of the dehydrodecalin moiety of **3** was tentatively assigned to be identical with **4** because **3** was considered as a biosynthetic precursor of **4** [20].

Compounds **1**‒**3** showed antimicrobial activity against *Kocuria rhizopila* with a MIC value of 6.5 μg/mL and **1** and **2** were also active against *Bacillus subtilis* with a MIC value of 12.5 μg/mL. Compounds **1**–**3** were inactive against *Staphylococcus aureus*, *Ralstonia solanacearum*, *Rhizobium radiobacter*, and *Candida albicans*. In addition, **1** and **2** exhibited cytotoxicity against P388 murine leukemia cells with IC_50_ of 33 and 89 μM, respectively.

## Conclusion

In summary, UV-based chemical screening of bioactive compounds from marine-derived actinomycetes led to the discovery of three new polyketides, nomimicins B (**1**), C (**2**), and D (**3**) along with a known congener, nomimicin A (**4**), from *Actinomadura* sp. AKA43. Compounds **1** and **2** are new members of the spirotetronate-class antibiotics, characterized by a macrocyclic structure containing a *trans*-decalin unit and a tetronic acid moiety spiro-linked with a cyclohexene ring. To date, more than 100 compounds [21–22], including tetrocarcin [23] and kijanimicin [24], are known within the spirotetronate class. Of these, nomimicins A–C (**4**, **1**, and **2**) are featured by the smallest macrocyclic ring and highly oxygenated dehydrodecalin moiety. Spirotetronates are known to be constructed by the intramolecular Diels–Alder reaction [20,25]. Compound **3** is very likely a biosynthetic precursor of **4**. This is the first report on the isolation of a biosynthetic precursor of spirotetronate antibiotics as an innate metabolite from a wild-type strain, while such an intermediate was previously obtained from a genetically engineered strain [26].

## Experimental

### General experimental procedures

Optical rotations were measured using a JASCO DIP-3000 polarimeter. ECD spectra were recorded on a JASCO J-720W spectropolarimeter. UV and IR spectra were recorded on a Shimadzu UV-1800 spectrophotometer and on a PerkinElmer Spectrum 100, respectively. All NMR experiments were performed on a Bruker AVANCE 500 spectrometer in CD_3_OD using the residual solvent proton (δ_Η_ 3.31) and carbon (δ_C_ 49.2) signals as internal standards. HRESITOFMS were recorded on a Bruker micrOTOF focus mass spectrometer. An Agilent HP1200 system equipped with a diode array detector was used for analysis and purification. The computational study was performed using MacroModel implemented in the Maestro 12.3 software package [27] and the Gaussian16, Revision C.01 program [28]. A part of these computations was conducted using the SuperComputer System, Institute for Chemical Research, Kyoto University. Molecular structures were visualized using Maestro 12.3 software package. ECD spectra were visualized using GaussView 6.0.16 and Microsoft Excel 2019.

### Microorganism

*Actinomadura* sp. AKA43 was isolated from a sea water sample collected from Sagami Bay at a depth of −800 m at the Izu-Akazawa DSW pumping station in Shizuoka, Japan, as previously reported [13]. The isolated strain was identified as a member of the genus *Actinomadura* on the basis of 100% similarity in the 16S rRNA gene sequence (1397 nucleotides, DDBJ accession number LC498623) with *Actinomadura geliboluensis* A8036^T^ (DDBJ accession number HQ157187).

### Fermentation

*Actinomadura* sp. AKA43 cultured on Bn-2 agar medium (soluble starch 0.5%, glucose 0.5%, meat extract (Kyokuto Pharmaceutical Industrial Co., Ltd.) 0.1%, yeast extract (Difco Laboratories) 0.1%, NZ-case (Wako Chemicals USA, Inc.) 0.2%, NaCl 0.2%, CaCO_3_ 0.1%, and agar 1.5% in distilled water of pH 7.0) was inoculated into a 500 mL K-1 ﬂask containing 100 mL of the V-22 seed medium (soluble starch 1%, glucose 0.5%, NZ-case 0.3%, yeast extract 0.2%, tryptone (Difco Laboratories) 0.5%, K_2_HPO_4_ 0.1%, MgSO_4_·7H_2_O 0.05%, and CaCO_3_ 0.3% in distilled water of pH 7.0). The ﬂask was shaken on a rotary shaker (200 rpm) at 30 °C for 4 days. For the production of nomimicins B (**1**) and C (**2**), the seed culture (3 mL) was transferred into 20 500 mL K-1 ﬂasks, each containing 100 mL of the A16 production medium (glucose 2%, Pharmamedia (Traders Protein) 1%, CaCO_3_ 0.5%, and Diaion^®^ HP-20 (Mitsubishi Chemical Corporation) 1% in distilled water of pH 7.0). The inoculated flasks were placed on a rotary shaker (200 rpm) at 30 °C for 7 days. For the production of nomimicin D (**3**), the seed culture (3 mL) was transferred into 20 500 mL K-1 ﬂasks, each containing 100 mL of the A11M production medium (glucose 0.2%, soluble starch 2.5%, yeast extract 0.5%, polypeptone (Wako Pure Chemical Industries, Ltd.) 0.5%, NZ-amine (Wako Pure Chemical Industries, Ltd.) 0.5%, CaCO_3_ 0.5%, and Diaion^®^ HP-20 1% in distilled water of pH 7.0). The inoculated flasks were placed on a rotary shaker (200 rpm) at 30 °C for 7 days.

### Isolation

At the end of the fermentation period, 100 mL of 1-butanol was added to each ﬂask, and the flasks were agitated on a rotary shaker for 1 h. The mixture was centrifuged at 6,000 rpm for 10 min, and the organic layer was separated from the aqueous layer containing the mycelium. Evaporation of the solvent gave 3.8 g of extract from 2 L of A16 culture. The extract was subjected to silica gel column chromatography with a step gradient of CHCl_3_/MeOH (1:0, 20:1, 10:1, 4:1, 2:1, 1:1, and 0:1, v/v). Fraction 4 (4:1) was concentrated to give 0.23 g of brown oil that was further purified by preparative HPLC (Cosmosil 5C18-ARII, 10 × 250 mm, 4 mL/min, UV detection at 254 nm) with 73% MeCN in 0.1% HCO_2_H solution to yield nomimicin A (**4**, 33 mg, *t*_R_ 21.5 min). Fractions 5 (2:1) and 6 (1:1) were combined and concentrated to provide 0.48 g of brown oil that was then fractionated by ODS column chromatography with a gradient of MeCN + 0.1% HCO_2_H solution (2:8, 3:7, 4:6, 5:5, 6:4, 7:3, and 8:2, v/v). The ODS fraction 6 (7:3) was concentrated to afford 0.22 g of semipure material. Final purification was achieved by preparative HPLC (Cosmosil 5C18-ARII, 10 × 250 mm, 4 mL/min, UV detection at 254 nm) with 52% MeCN in 0.1% HCO_2_H solution to yield nomimicin B (**1**, 16.1 mg, *t*_R_ 19.5 min) and nomimicin C (**2**, 12.2 mg, *t*_R_ 21.5 min). Similarly, evaporation of the solvent gave 3.0 g of extract from 2 L of A11M culture. The extract was subjected to silica gel column chromatography with a gradient of CHCl_3_/MeOH (1:0, 20:1, 10:1, 4:1, 2:1, 1:1, and 0:1, v/v). Fraction 7 (0:1) was concentrated to give 0.37 g of brown oil, which was then fractionated by ODS column chromatography with a gradient of MeCN + 0.1% HCO_2_H solution (2:8, 3:7, 4:6, 5:5, 6:4, 7:3, and 8:2, v/v). The ODS fraction 5 (6:4) was concentrated to give 72.6 mg of semipure material. Final purification using preparative HPLC (Cosmosil XTerra Prep RP18, 10 × 250 mm, 4 mL/min, UV detection at 254 nm) with 38% MeCN in 10 mM NH_4_HCO_3_ solution yielded nomimicin D (**3**, 6.0 mg, *t*_R_ 30.5 min).

### Nomimicin B (**1**)

Colorless amorphous solid; [α]_D_^23^ −29 (*c* 0.10, MeOH); UV (MeOH) λ_max_ (log ε) 246 (3.83), 293 nm (3.71); ECD (*c* 9.5 × 10^−5^, MeOH) λ_ext_ (Δε) 208 (−5.27), 247 (+3.72), 294 nm (−1.24); IR ν_max_: 3360, 2965, 1755, 1619, 1408, 1088, 998 cm^−1^; see Table 1 for ^1^H and ^13^C NMR data; HRESITOFMS (*m*/*z*): [M + Na]^+^ calcd for C_30_H_40_O_8_Na, 551.2615; found, 551.2612.

### Nomimicin C (**2**)

Colorless amorphous solid; [α]_D_^23^ −12 (*c* 0.10, MeOH); UV (MeOH) λ_max_ (log ε) 246 (3.93), 292 nm (3.78); ECD (*c* 9.7 × 10^−5^, MeOH) λ_ext_ (Δε) 208 (−6.63), 246 (+4.08), 298 nm (−1.39); IR ν_max_: 3380, 2963, 1744, 1618, 1404, 1097, 1007 cm^−1^; see Table 1 for ^1^H and ^13^C NMR data; HRESITOFMS (*m*/*z*): [M + Na]^+^ calcd for C_30_H_40_O_7_Na, 535.2666; found, 535.2665.

### Nomimicin D (**3**)

Colorless amorphous solid; [α]_D_^23^ −70 (*c* 0.10. MeOH); UV (MeOH) λ_max_ (log ε) 243 (3.99), 302 nm (3.54); IR ν_max_: 3380, 2963, 1723, 1619, 1413, 1258, 1010 cm^−1^; see Table 2 for ^1^H and ^13^C NMR data; HRESITOFMS (*m*/*z*): [M + Na]^+^ calcd for C_30_H_40_O_6_Na, 519.2717; found, 519.2717.

### Nomimicin A (**4**)

Colorless amorphous solid; [α]_D_^23^ −87 (*c* 0.10. CHCl_3_, lit. [α]_D_^23^ −94 (*c* 0.10. CHCl_3_) [15]); UV (MeOH) λ_max_ (log ε) 242 (3.73), 298 nm (3.59); ECD (*c* 1.0 × 10^−4^, MeOH) λ_ext_ (Δε) 208 (−4.98), 244 (+3.85), 298 nm (−1.09); IR ν_max_: 3348, 2938, 1735, 1620, 1435, 997 cm^−1^; HRESITOFMS (*m*/*z*): [M + Na]^+^ calcd for C_30_H_40_O_6_Na, 519.2717; found, 519.2722.

### ECD calculations

The conformational sampling of structure **4a** was performed by applying 100,000 steps of the Monte Carlo Multiple Minimum (MCMM) method with PRCG energy minimization by the OPLS3e force field to obtain 56 conformational isomers within 10.0 kcal/mol from the minimum energy conformer. Geometries of the conformers were then optimized at the M06-2X/6-31G(d) level of theory with the SMD solvation model. Frequency calculations were carried out at the same level of theory to confirm the absence of imaginary frequencies and obtain thermal corrections for the Gibbs free energy. After eliminating duplicated structures with the threshold of 0.01 Å RMSD, the single-point energy was calculated at the M06-2X/def2-TZVP-SMD level of theory, affording 24 conformers within 3.0 kcal/mol from the minimum Gibbs free energy. The ECD spectrum of each conformer was simulated by the TDDFT calculation of 25 excited states at the ωB97X-D/def2-TZVP-PCM level of theory. The spectrum of structure **4a** was created by the weighted average of the above-obtained spectra (half-width: 0.29 eV) according to the Boltzmann distribution, corrected by a red-shift of 15 nm, and scaled to adjust the strength of the vertical axis. The spectra of structures **4b**, **4c**, and **4d** were similarly simulated using 122, 126, and 475 OPLS3e-minimized structures and 26, 22, and 31 DFT-optimized structures, respectively. The spectrum of **4** was created using the weighted average of the spectra of all low-lying conformers of **4a**–**d** according to the respective Boltzmann distribution. The Cartesian coordinates and the energies of the most stable conformers for each tautomer are included in Supporting Information File 1.

### Biological assays

Antimicrobial activity and cytotoxicity were evaluated according to the procedures previously described [29].

## Supporting Information

File 1 Copies of UV, IR, and NMR spectra for **1**–**4** as well as Cartesian coordinates and energies of the most stable conformers of **4a**–**d**.
