# Supplementary material for: Nomimicins B–D, new tetronate-class polyketides from a marine-derived actinomycete of the genus Actinomadura
Source: Beilstein J Org Chem. 2021 Aug 27;17:2194–202. doi: 10.3762/bjoc.17.141 (PMC8404215; doi:10.3762/bjoc.17.141)
Supplement: File 1 — Copies of UV, IR, and NMR spectra for 1–4 as well as Cartesian coordinates and energies of the most stable conformers of 4a–d. [file Beilstein_J_Org_Chem-17-2194-s001.pdf]

BEILSTEIN JOURNAL OF ORGANIC CHEMISTRY

Supporting Information

for

Nomimicins B–D, new tetronate-class polyketides from a marine-derived actinomycete of the genus *Actinomadura*

Zhiwei Zhang, Tao Zhou, Taehui Yang, Keisuke Fukaya, Enjuro Harunari, Shun Saito, Katsuhisa Yamada, Chiaki Imada, Daisuke Urabe and Yasuhiro Igarashi

Beilstein J. Org. Chem. **2021,** *17,* 2194–2202. doi:10.3762/bjoc.17.141

Copies of UV, IR, and NMR spectra for 1–4 as well as Cartesian coordinates and energies of the most stable conformers of 4a–d

License and Terms: This is a supporting information file under the terms of the Creative Commons Attribution License (https://creativecommons.org/licenses/by/4.0). Please note that the reuse, redistribution and reproduction in particular requires that the author(s) and source are credited and that individual graphics may be subject to special legal provisions.
The license is subject to the *Beilstein Journal of Organic Chemistry* terms and conditions: (https://www.beilstein-journals.org/bjoc/terms)

Table of Content

Figure S1. UV spectrum of nomimicin B (**1**). S3
Figure S2. IR spectrum of **1**........ S4
Figure S3. ^{1}H NMR spectrum of **1** (500 MHz, CD_3OD). S5
Figure S4. ^{13}C NMR spectrum of **1** (125 MHz, CD_3OD). S6
Figure S5. COSY spectrum of **1** (500 MHz, CD_3OD)........ S7
Figure S6. HSQC spectrum of **1** (500 MHz, CD_3OD)........ S8
Figure S7. HMBC spectrum of **1** (500 MHz, CD_3OD). S9
Figure S8. NOESY spectrum of **1** (500 MHz, CD_3OD). S10
Figure S9. ROESY spectrum of **1** (500 MHz, CD_3OD). S11
Figure S10. UV spectrum of nomimicin C (**2**). S12
Figure S11. IR spectrum of **2**........ S13
Figure S12. ^{1}H NMR spectrum of **2** (500 MHz, CD_3OD). S14
Figure S13. ^{13}C NMR spectrum of **2** (125 MHz, CD_3OD). S15
Figure S14. COSY spectrum of **2** (500 MHz, CD_3OD)........ S16
Figure S15. HSQC spectrum of **2** (500 MHz, CD_3OD)........ S17
Figure S16. HMBC spectrum of **2** (500 MHz, CD_3OD)........ S18
Figure S17. NOESY spectrum of **2** (500 MHz, CD_3OD). S19
Figure S18. ROESY spectrum of **2** (500 MHz, CD_3OD). S20
Figure S19. UV spectrum of nomimicin D (**3**). S21
Figure S20. IR spectrum of **3**........ S22
Figure S21. ^{1}H NMR spectrum of **3** (500 MHz, CD_3OD). S23
Figure S22. ^{13}C NMR spectrum of **3** (125 MHz, CD_3OD). S24
Figure S23. COSY spectrum of **3** (500 MHz, CD_3OD)........ S25
Figure S24. HSQC spectrum of **3** (500 MHz, CD_3OD)........ S26
Figure S25. HMBC spectrum of **3** (500 MHz, CD_3OD)........ S27
Figure S26. NOESY spectrum of **3** (500 MHz, CD_3OD). S28
Figure S27. ROESY spectrum of **3** (500 MHz, CD_3OD). S29
Figure S28. UV spectrum of nomimicin (**4**). S30
Figure S29. IR spectrum of **4**........ S31
Figure S30. ^{1}H NMR spectrum of **4** (500 MHz, CD_3OD). S32
Figure S31. ^{13}C NMR spectrum of **4** (125 MHz, CD_3OD). S33
Figure S32. COSY spectrum of **4** (500 MHz, CD_3OD)........ S34
Figure S33. HSQC spectrum of **4** (500 MHz, CD_3OD)........ S35
Figure S34. HMBC spectrum of **4** (500 MHz, CD_3OD)........ S36

Figure S35. COSY and key HMBC correlations for **2**. S37
Figure S36. Relative correlations for **2** determined by ROESY analysis. S37
Table S1. NOESY and ROESY correlations of nomimicin B (**1**). S38
Table S2. NOESY and ROESY correlations of nomimicin C (**2**). S39
Table S3. ROESY and NOESY correlations of nomimicin D (**3**). S40
Table S4. Cartesian coordinates and energies of the most stable conformer of **4a**........ S41
Table S5. Cartesian coordinates and energies of the most stable conformer of **4b**. S43
Table S6. Cartesian coordinates and energies of the most stable conformer of **4c**. S45
Table S7. Cartesian coordinates and energies of the most stable conformer of **4d**. S47

Figure S1. UV spectrum of nomimicin B (**1**).

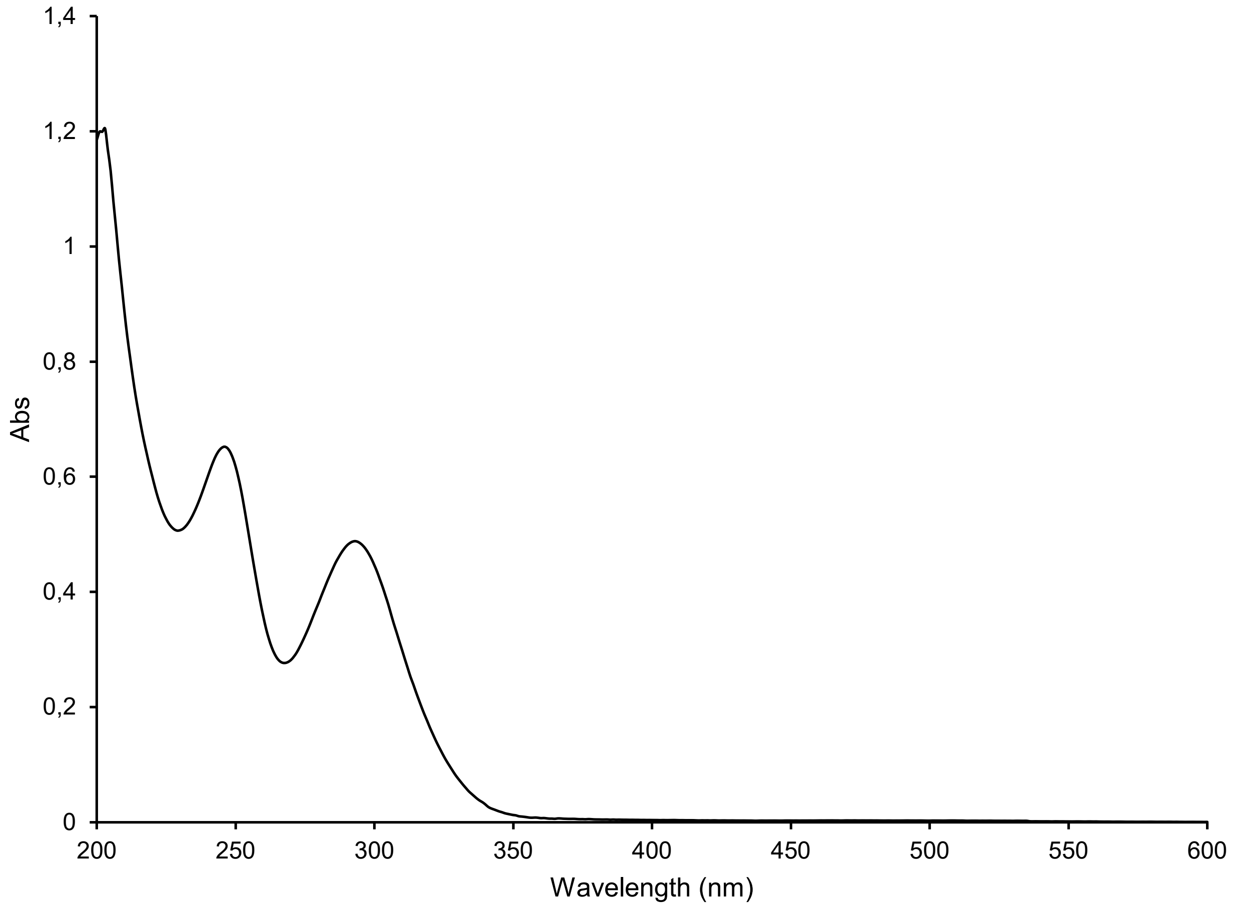

Figure S2. IR spectrum of **1.**

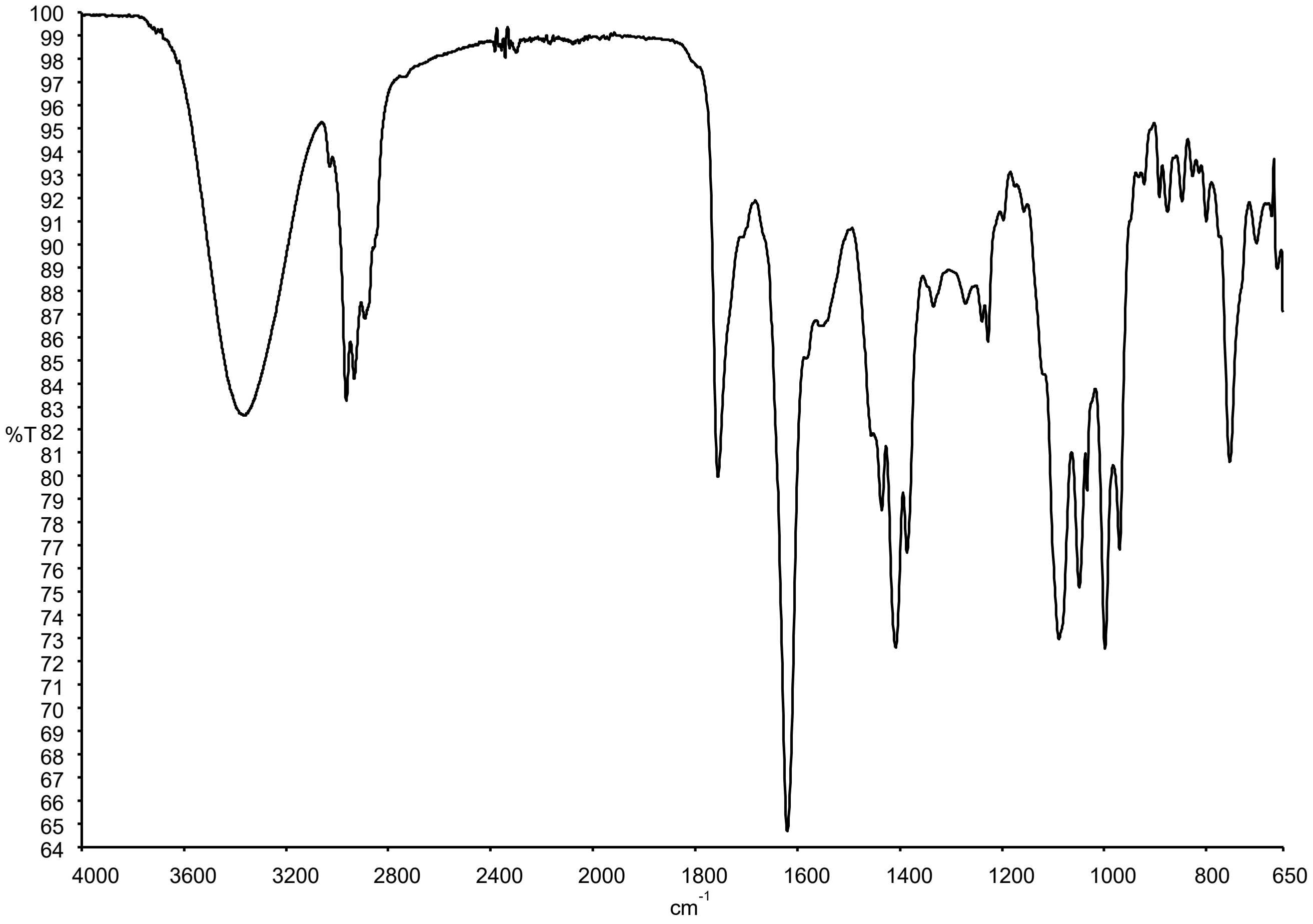

Figure S3. ^{1}H NMR spectrum of **1** (500 MHz, CD_3OD).

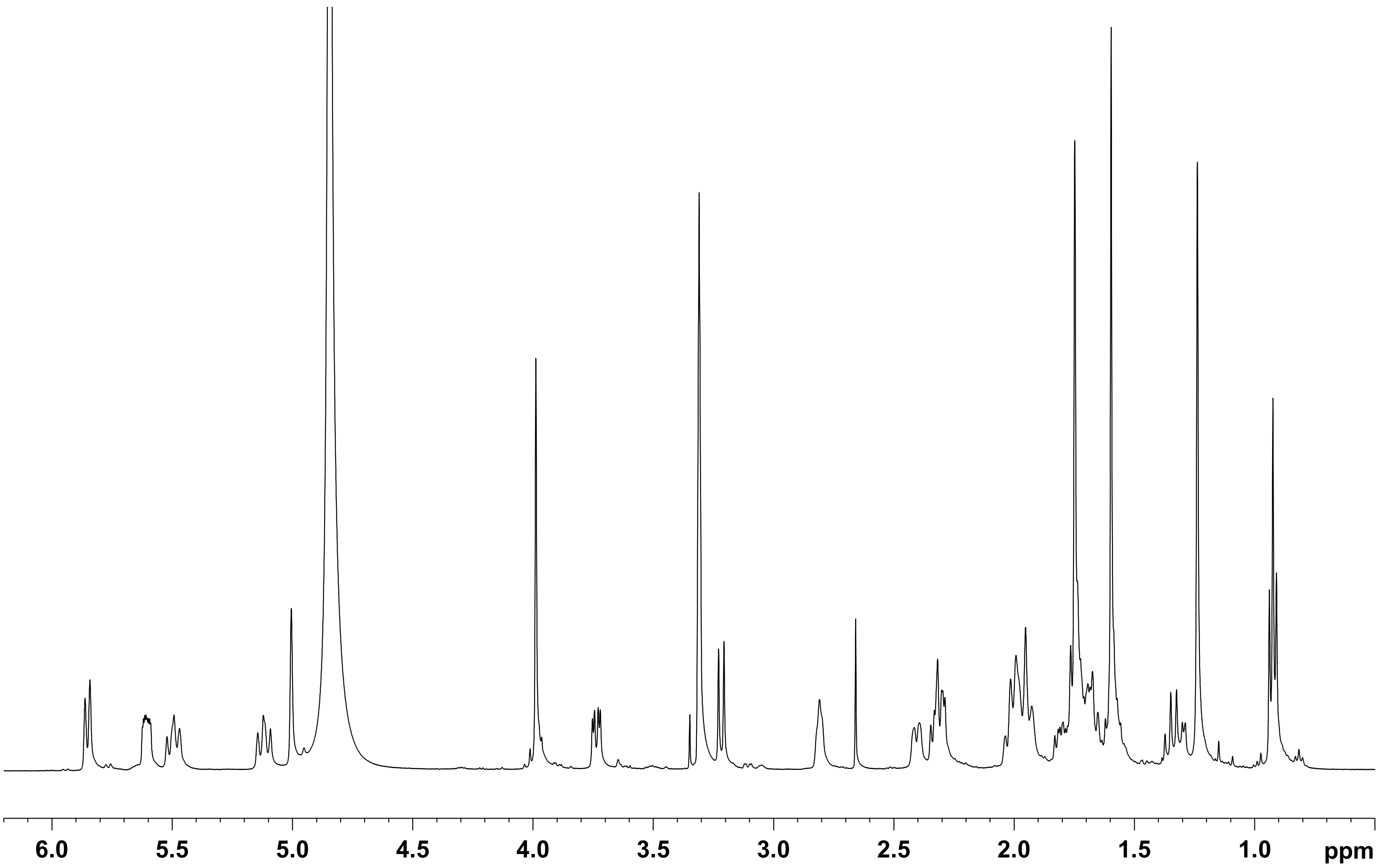

Figure S4. ^{13}C NMR spectrum of **1** (125 MHz, CD_3OD).

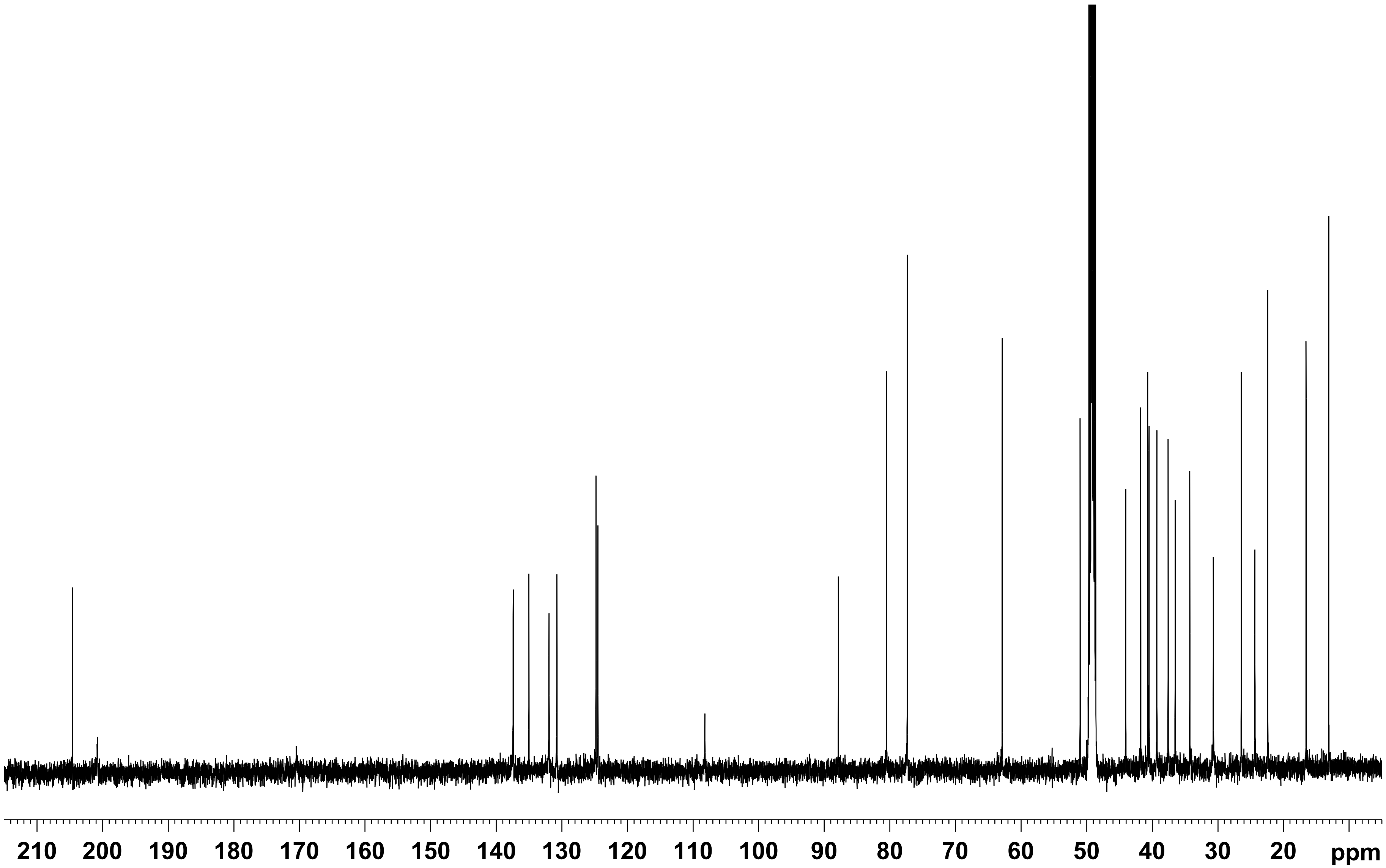

Figure S5. COSY spectrum of **1** (500 MHz, CD_3OD).

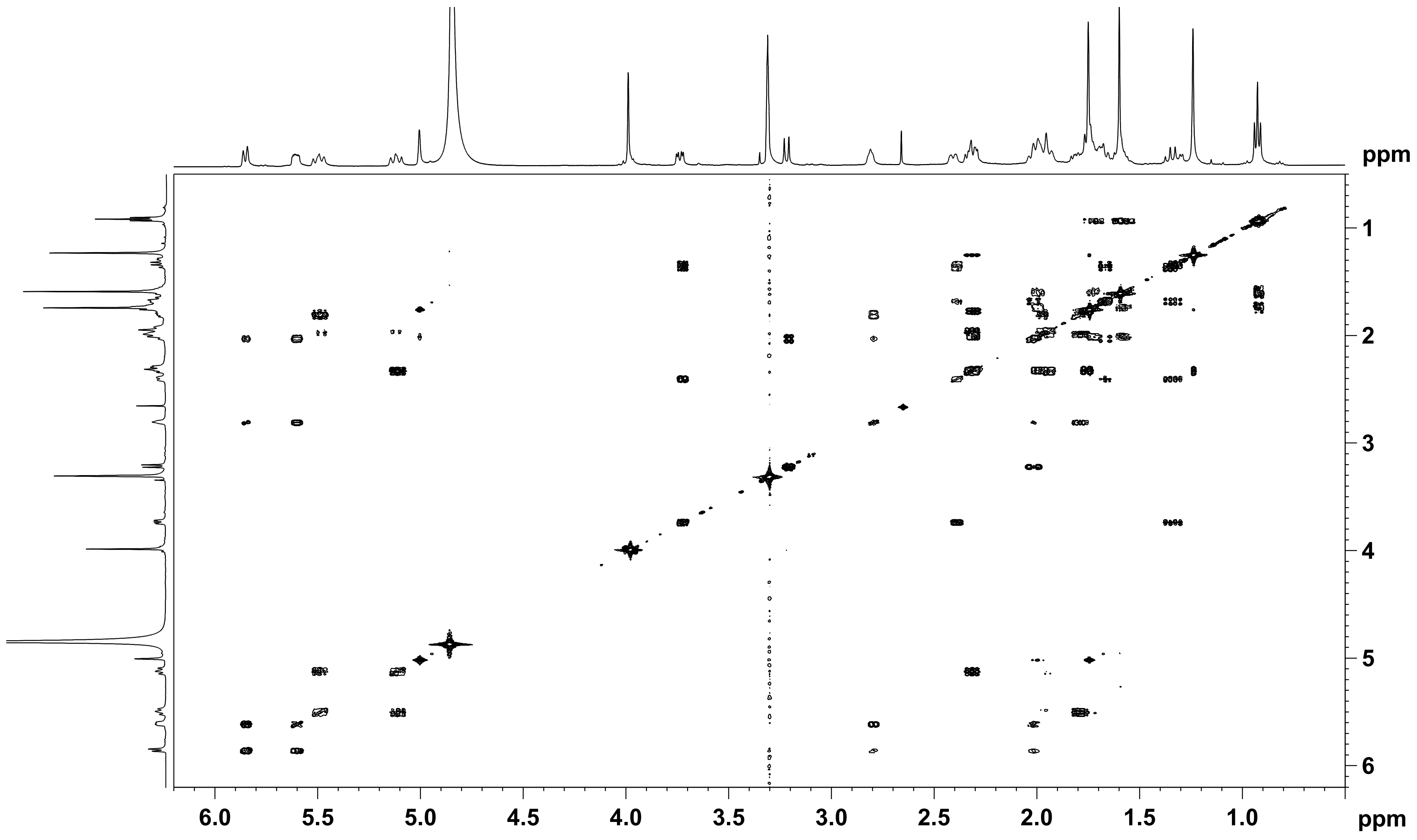

Figure S6. HSQC spectrum of **1** (500 MHz, CD_3OD).

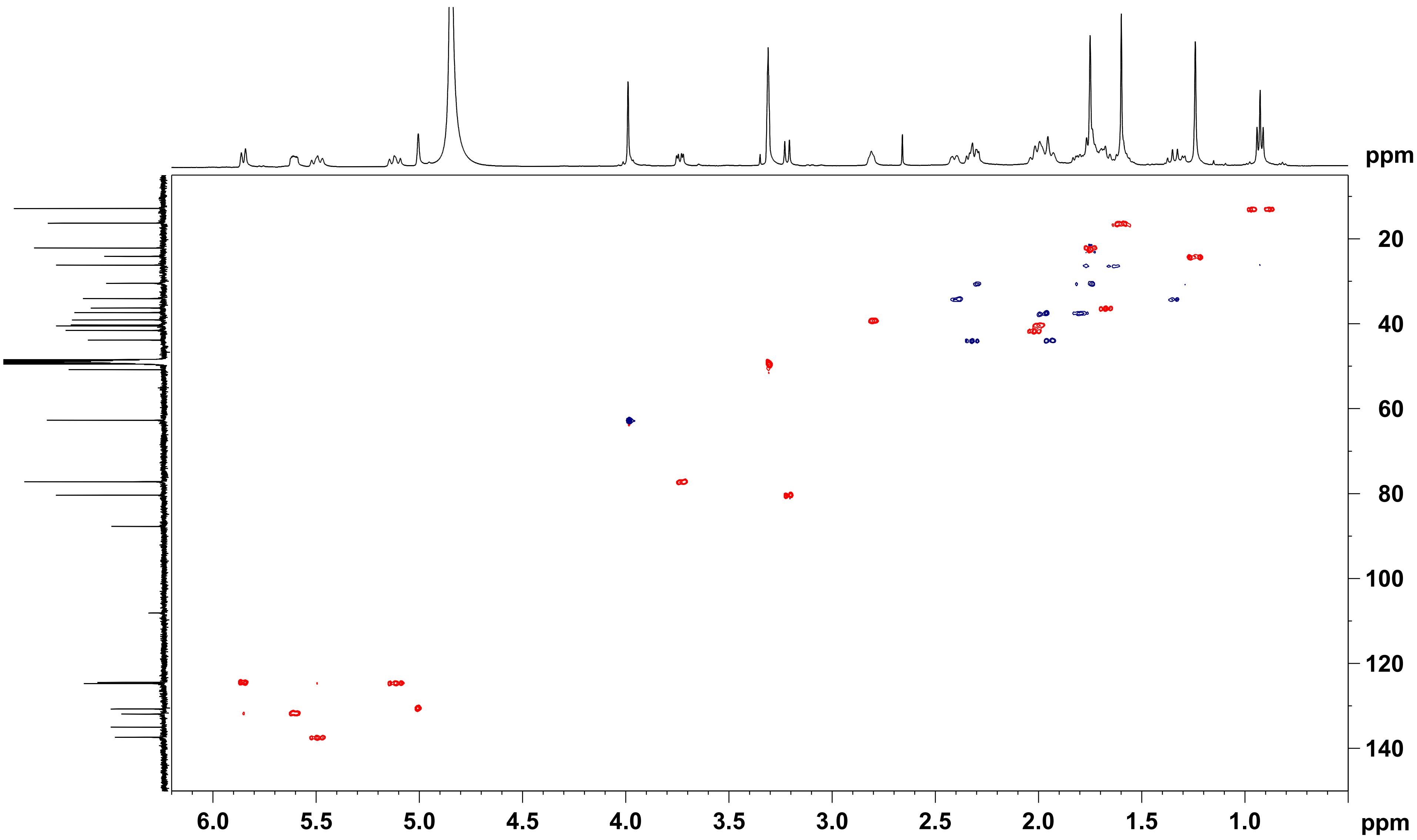

Figure S7. HMBC spectrum of **1** (500 MHz, CD_3OD).

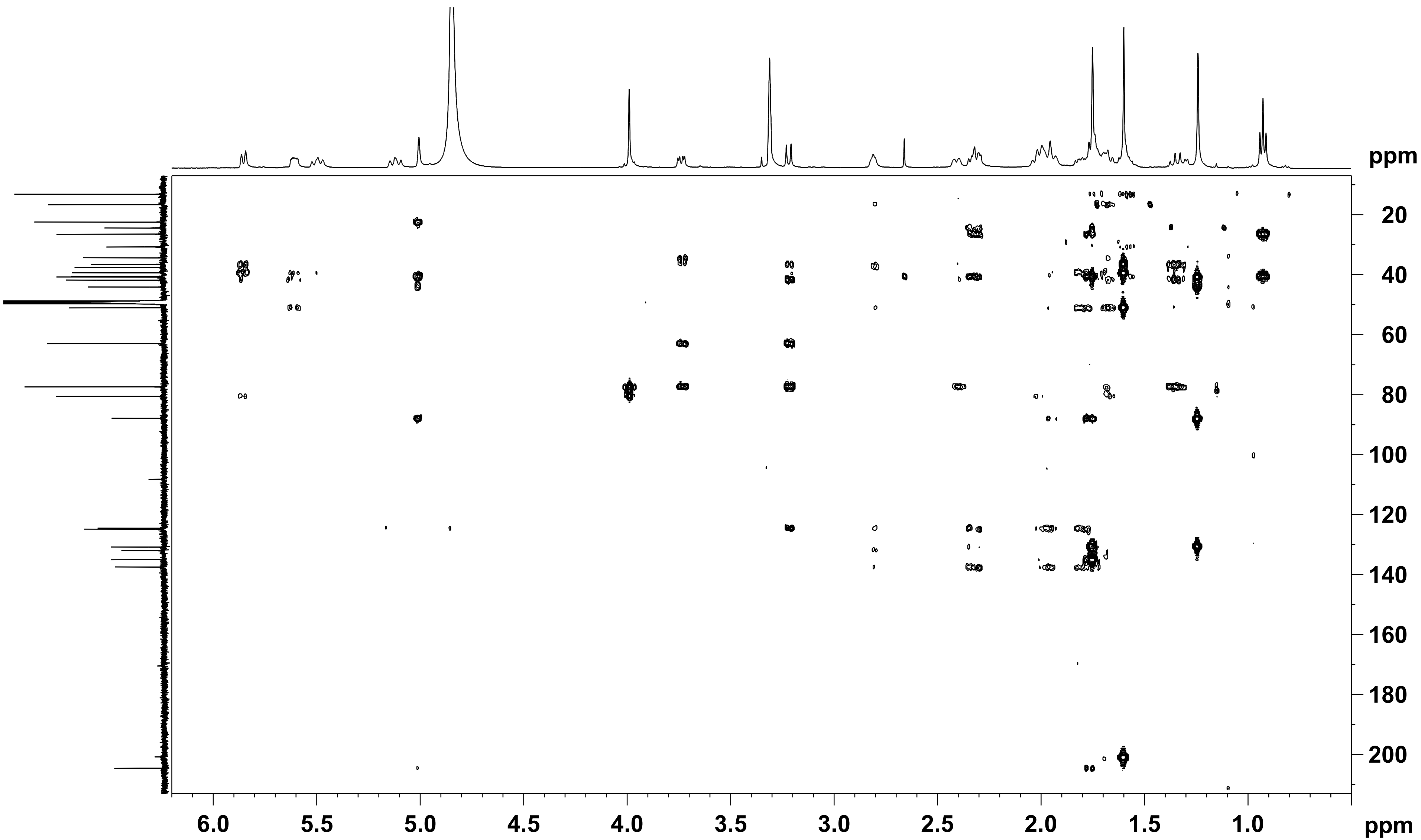

Figure S8. NOESY spectrum of **1** (500 MHz, CD_3OD).

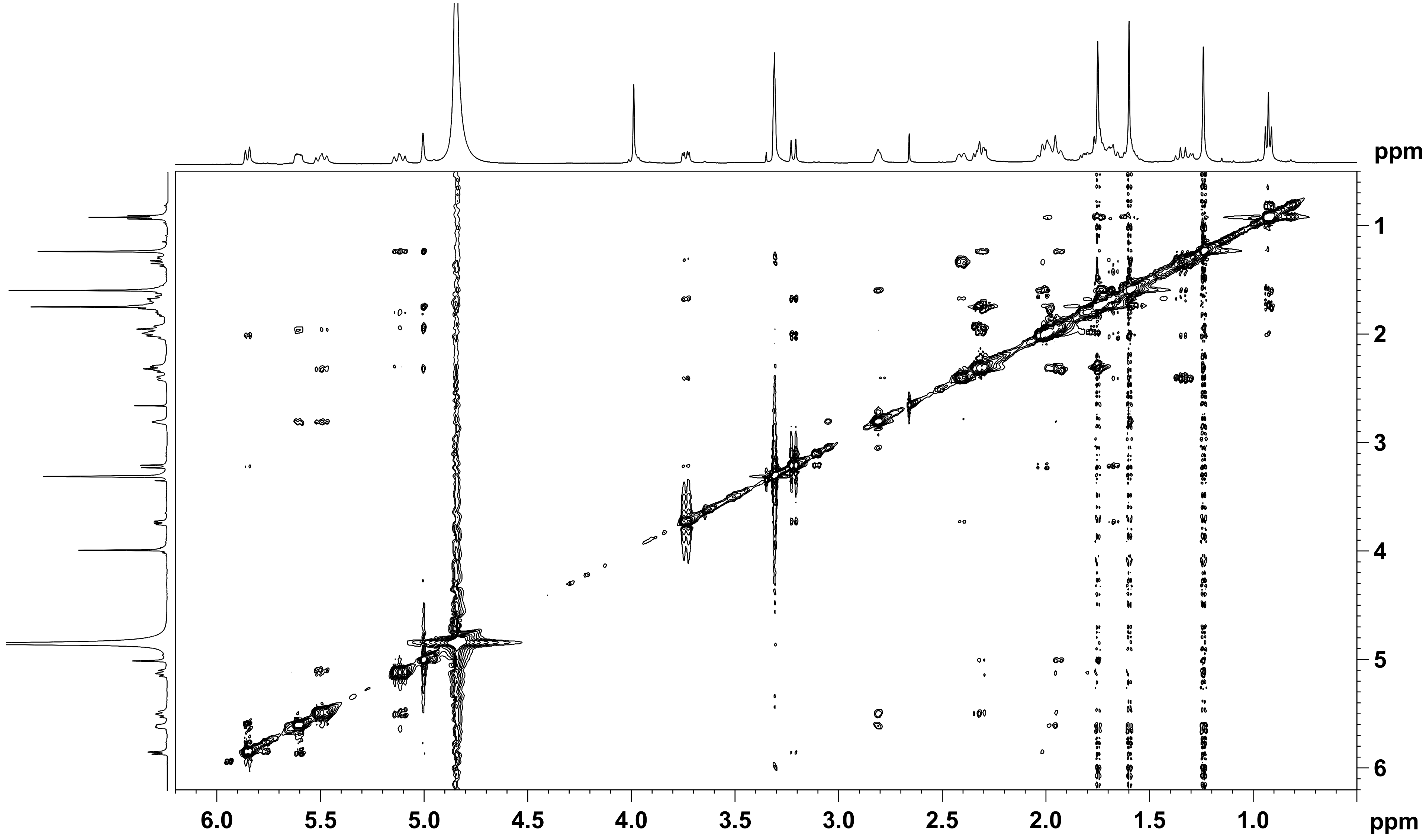

Figure S9. ROESY spectrum of **1** (500 MHz, CD_3OD).

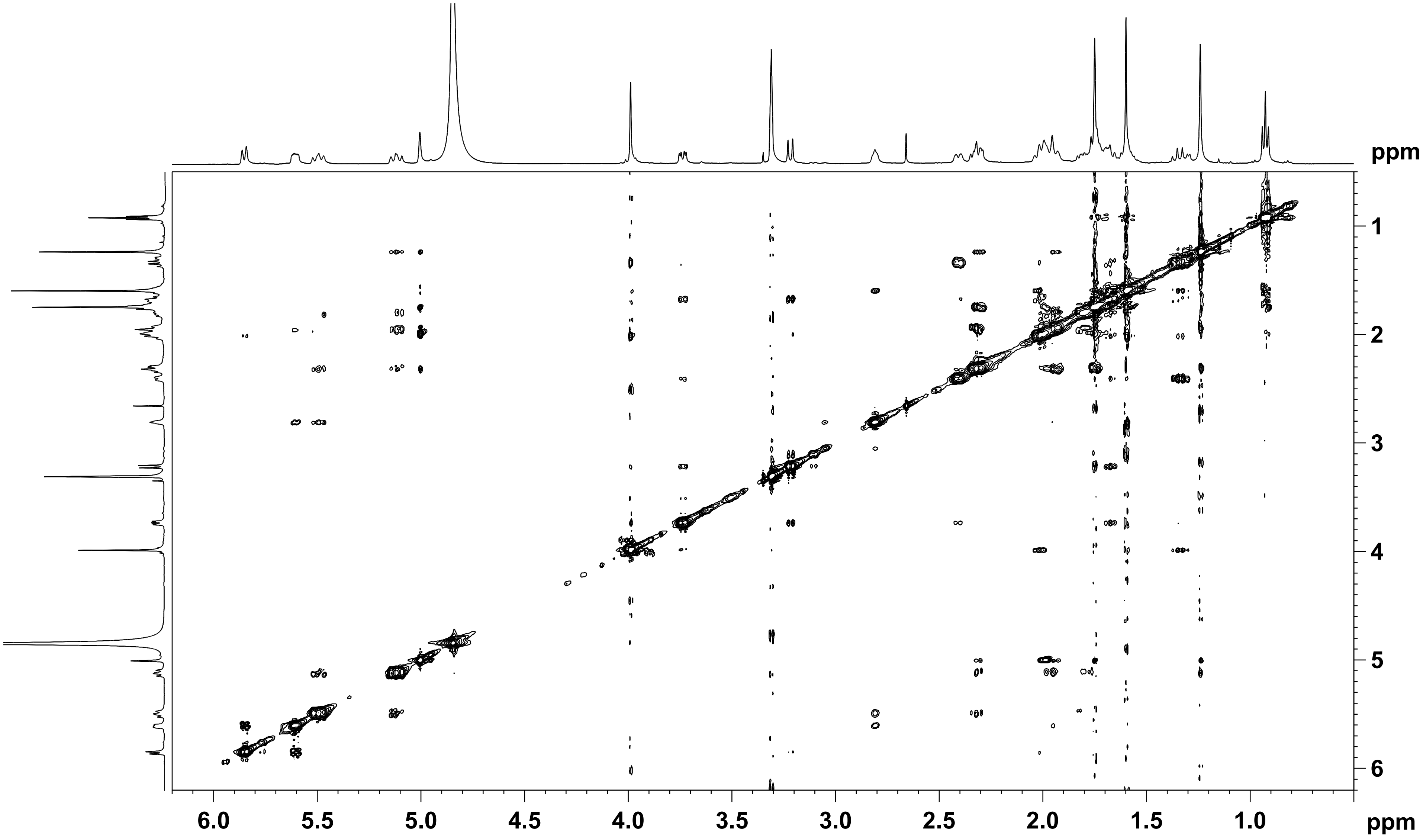

Figure S10. UV spectrum of nomimicin C (**2**).

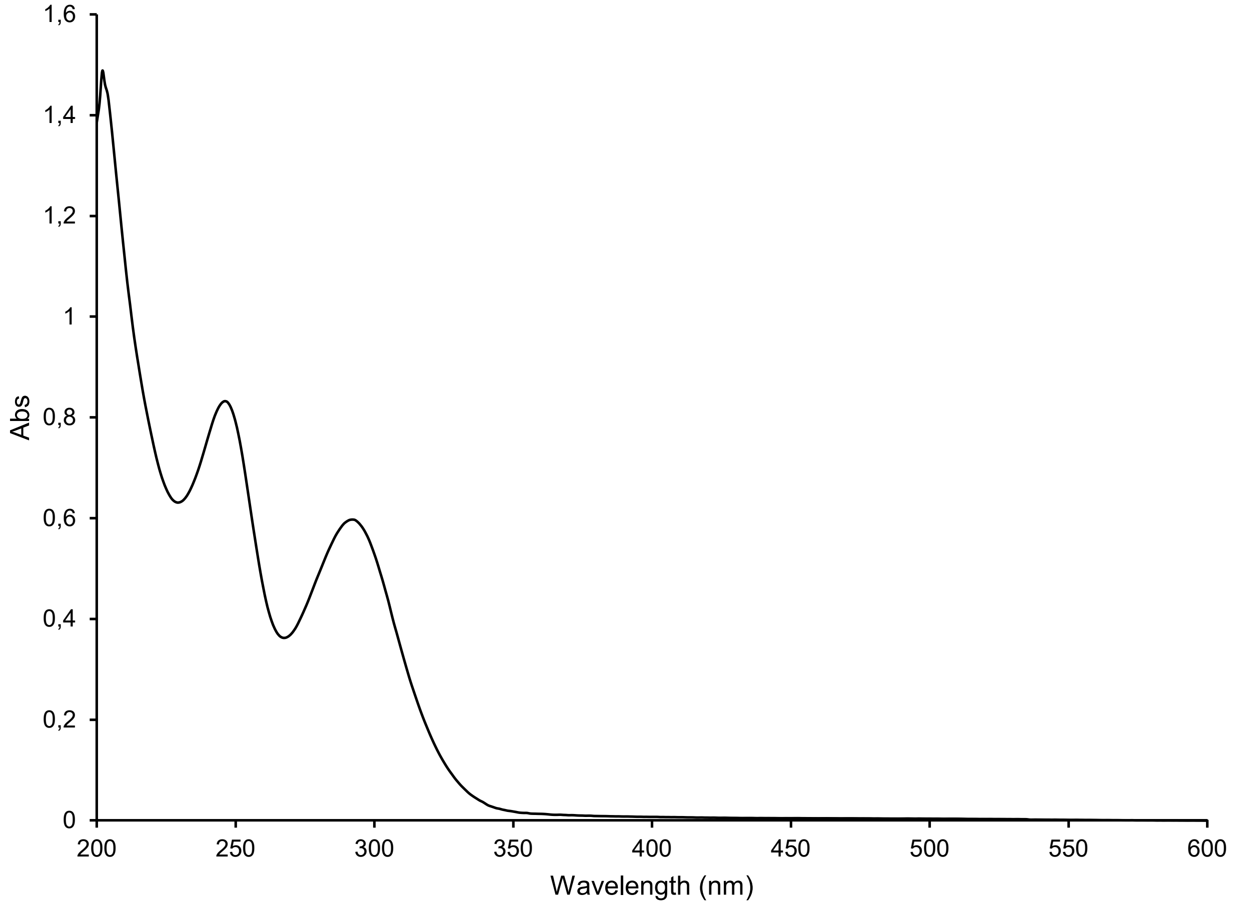

Figure S11. IR spectrum of **2.**

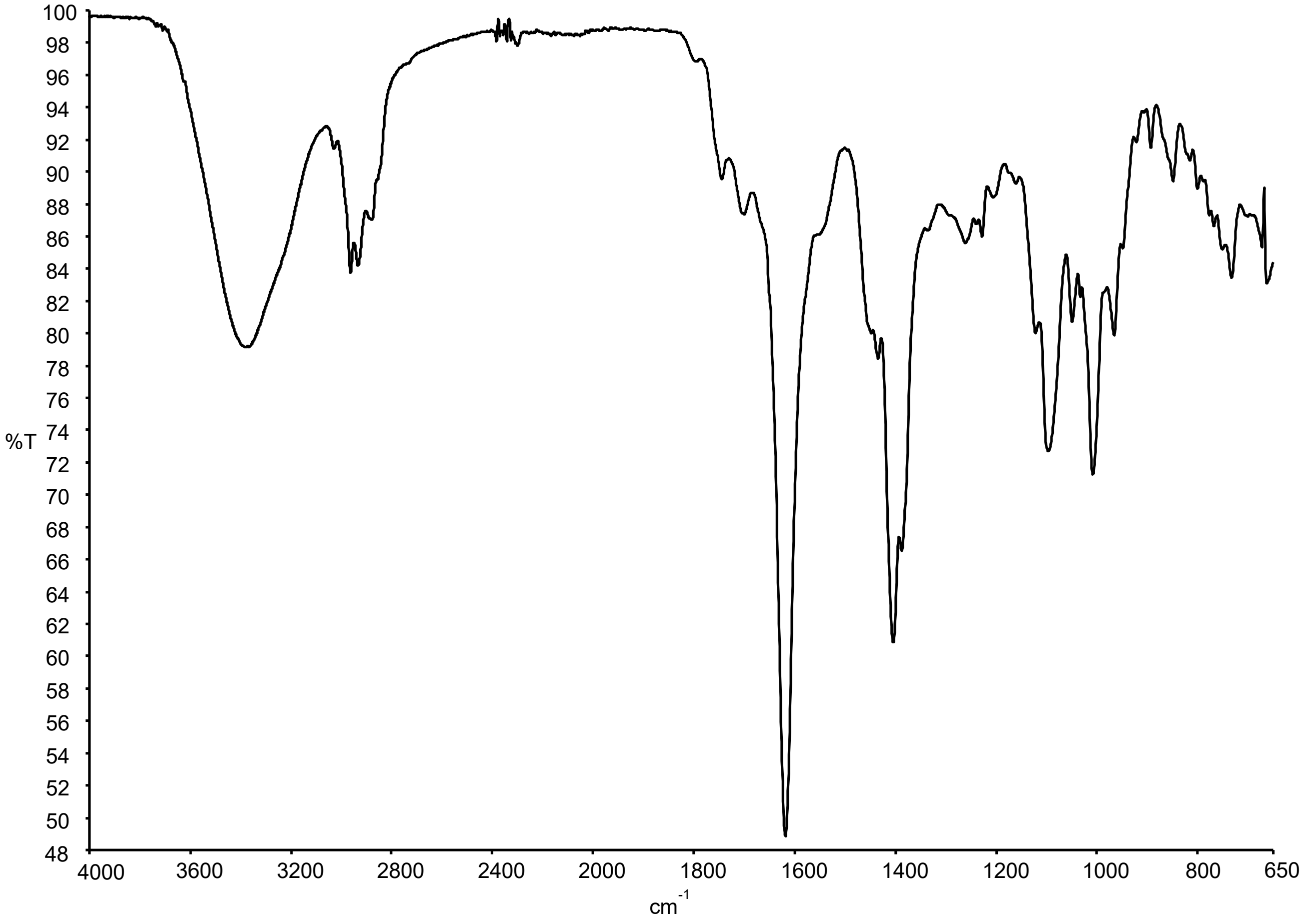

Figure S12. ^{1}H NMR spectrum of **2** (500 MHz, CD_3OD).

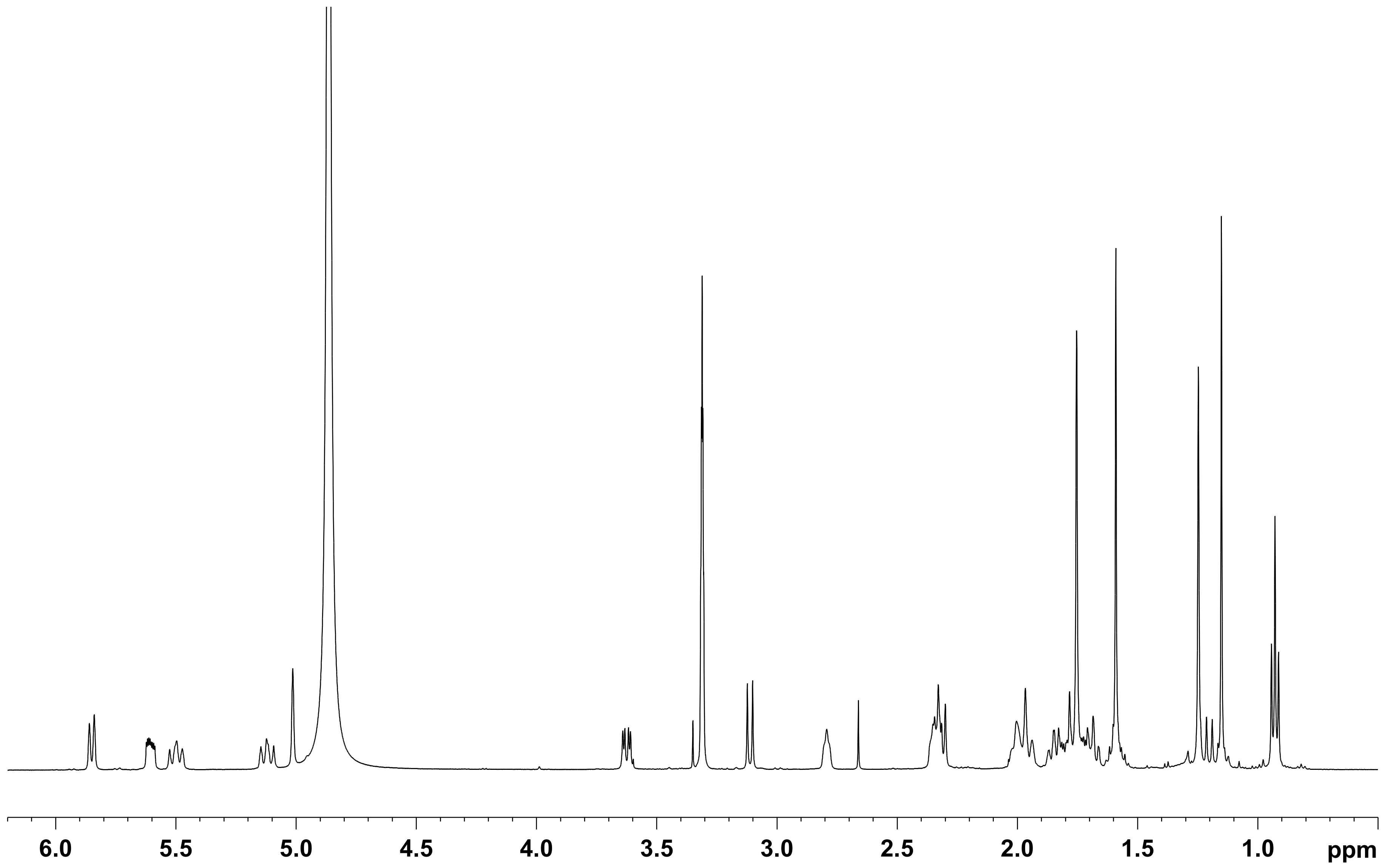

Figure S13. ^{13}C NMR spectrum of **2** (125 MHz, CD_3OD).

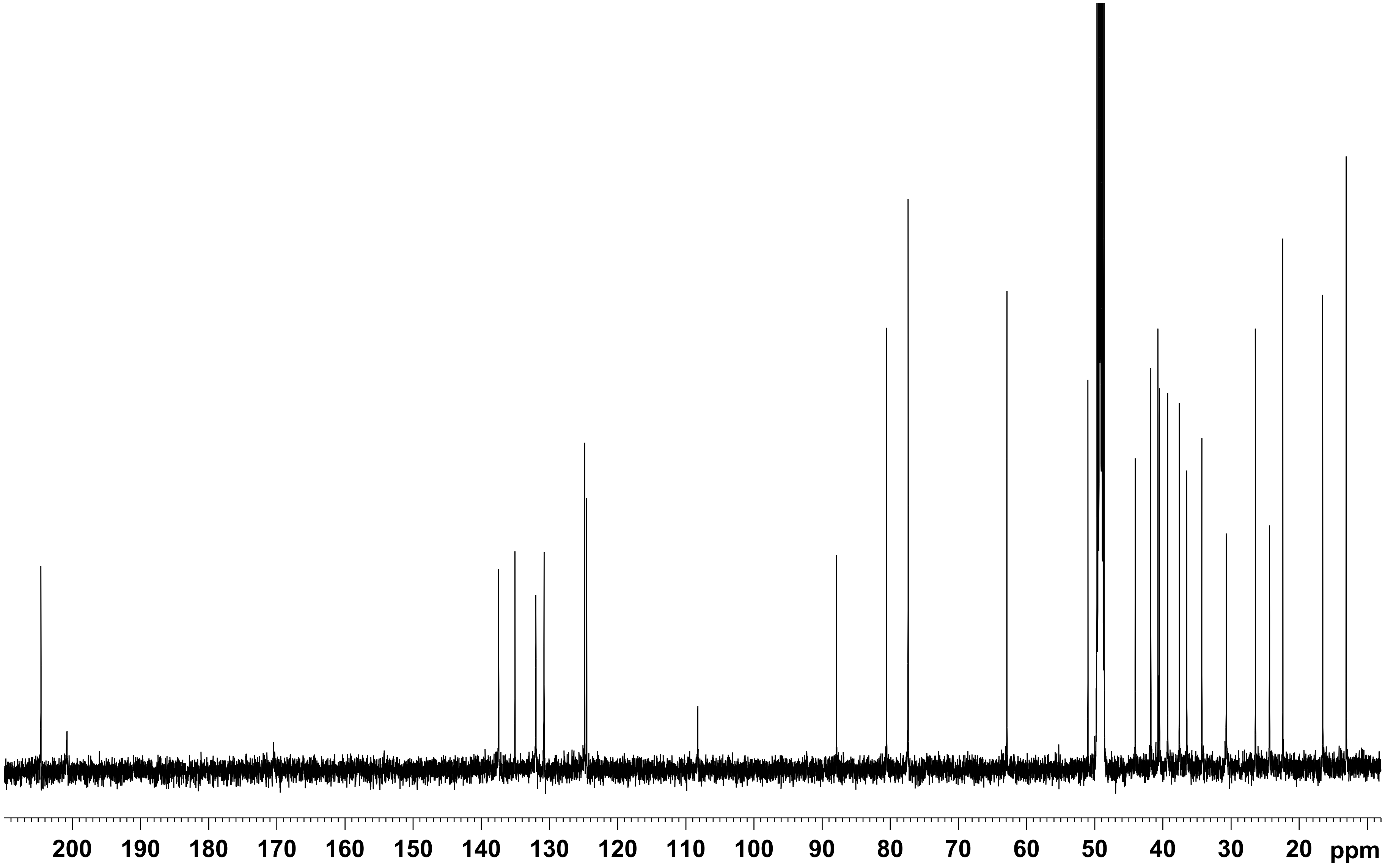

Figure S14. COSY spectrum of **2** (500 MHz, CD_3OD).

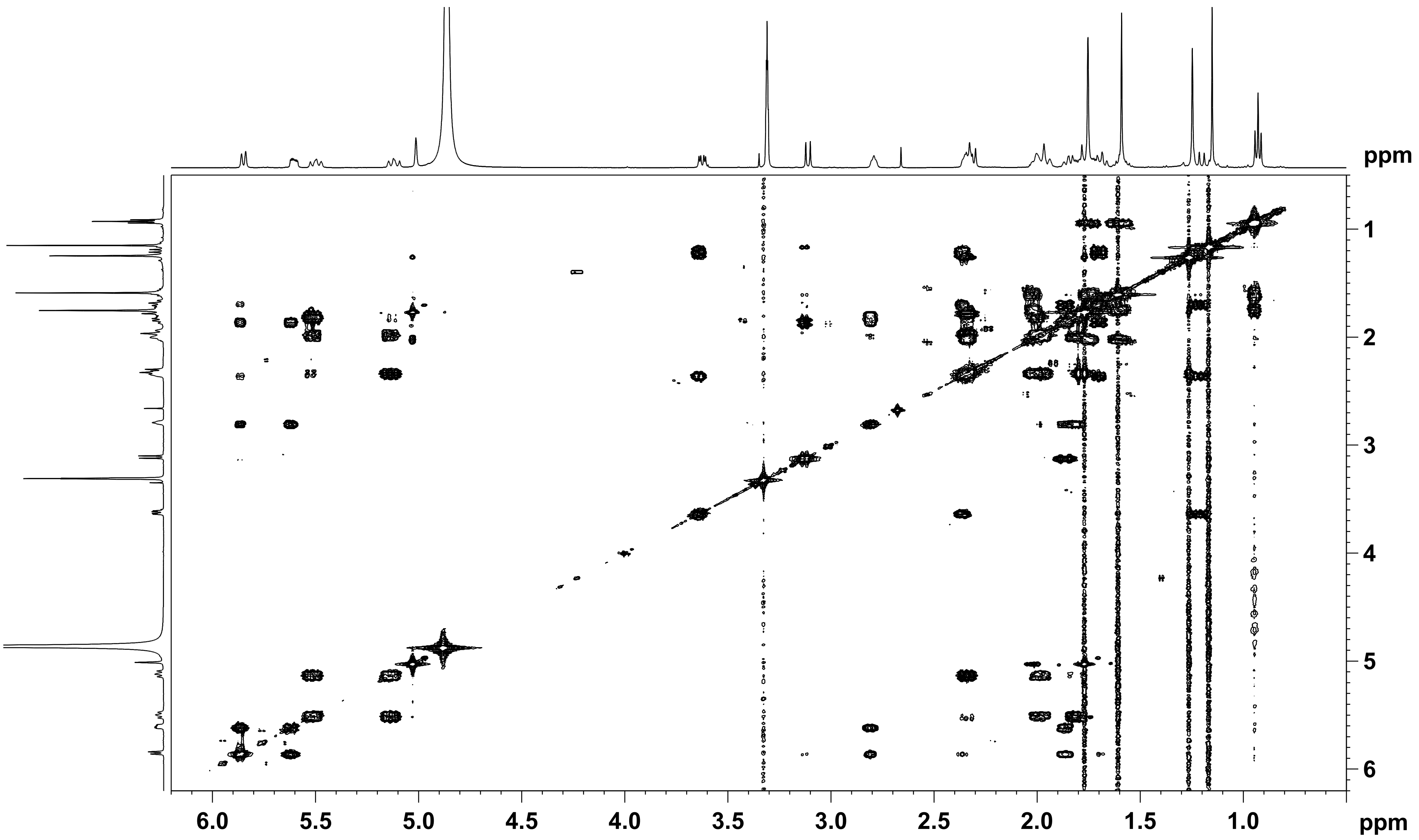

Figure S15. HSQC spectrum of **2** (500 MHz, CD_3OD).

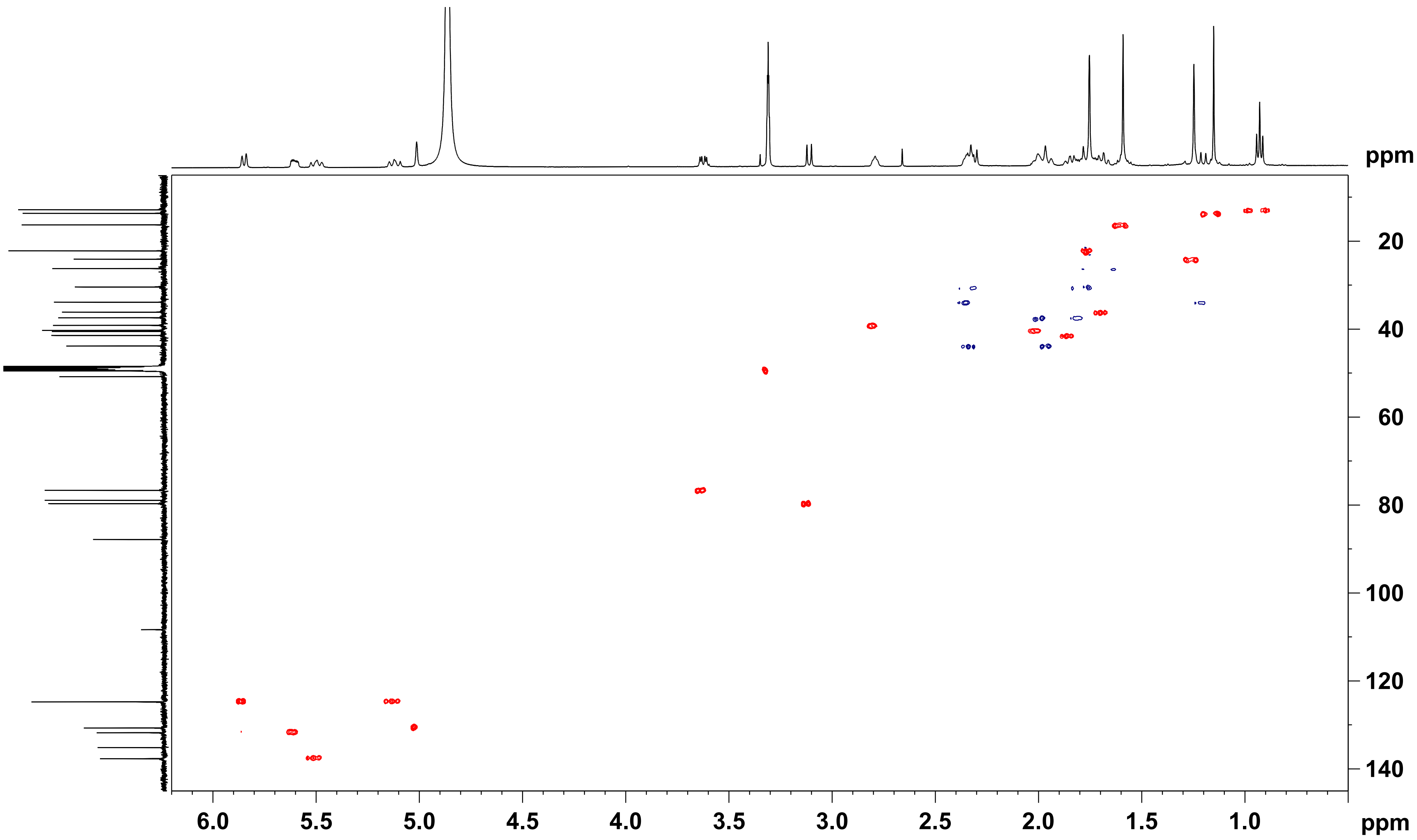

Figure S16. HMBC spectrum of **2** (500 MHz, CD_3OD).

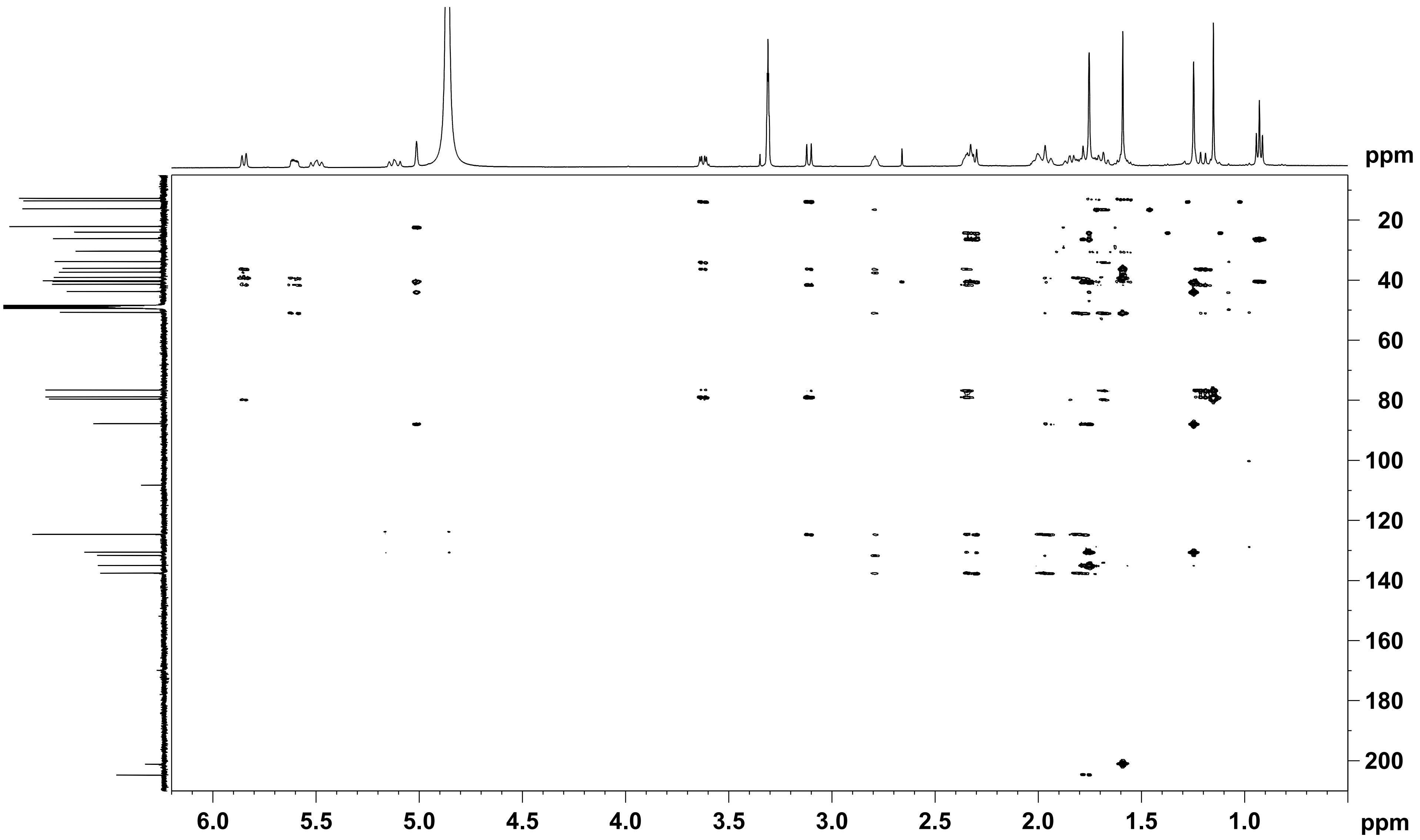

Figure S17. NOESY spectrum of **2** (500 MHz, CD_3OD).

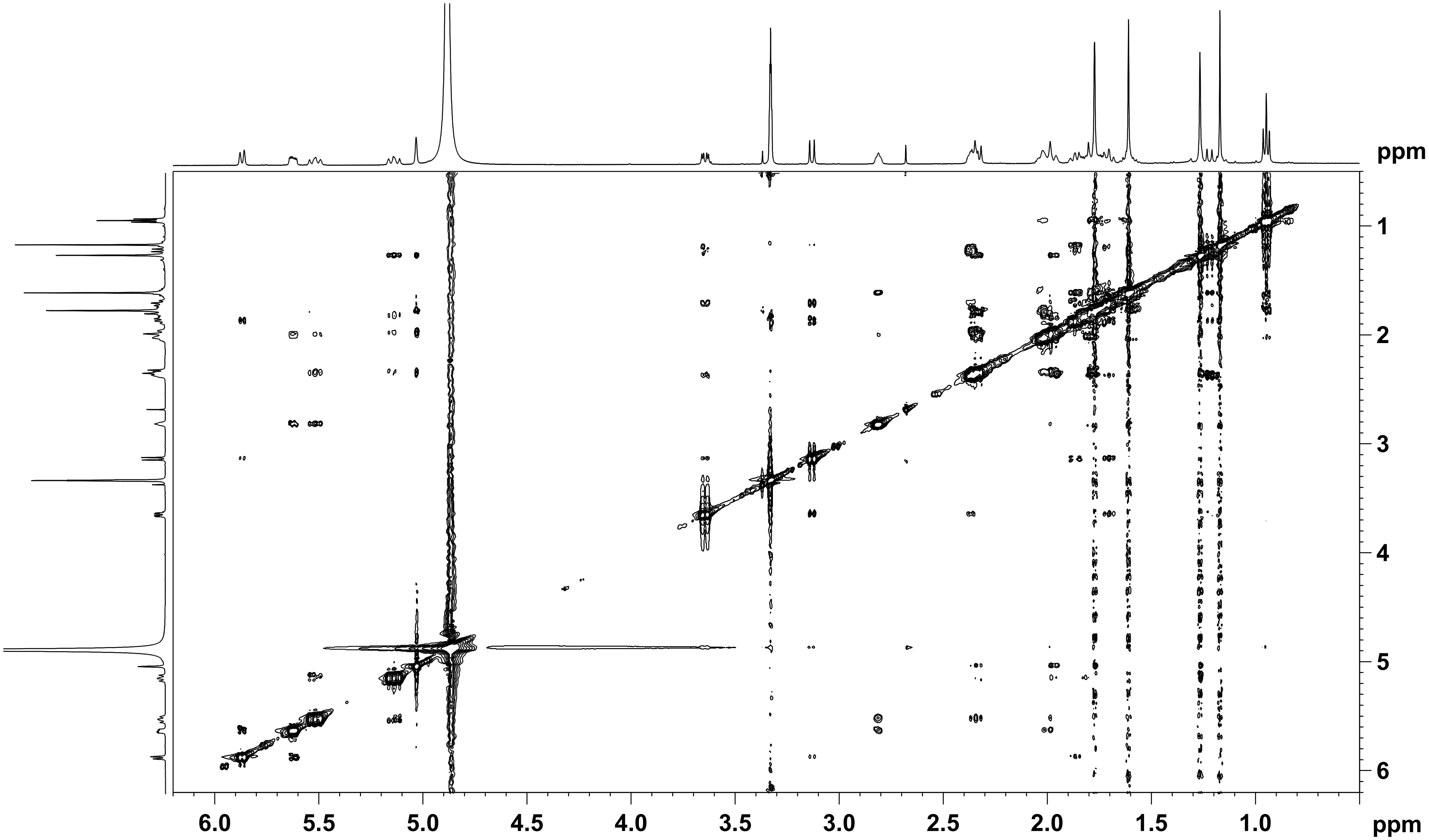

Figure S18. ROESY spectrum of **2** (500 MHz, CD_3OD).

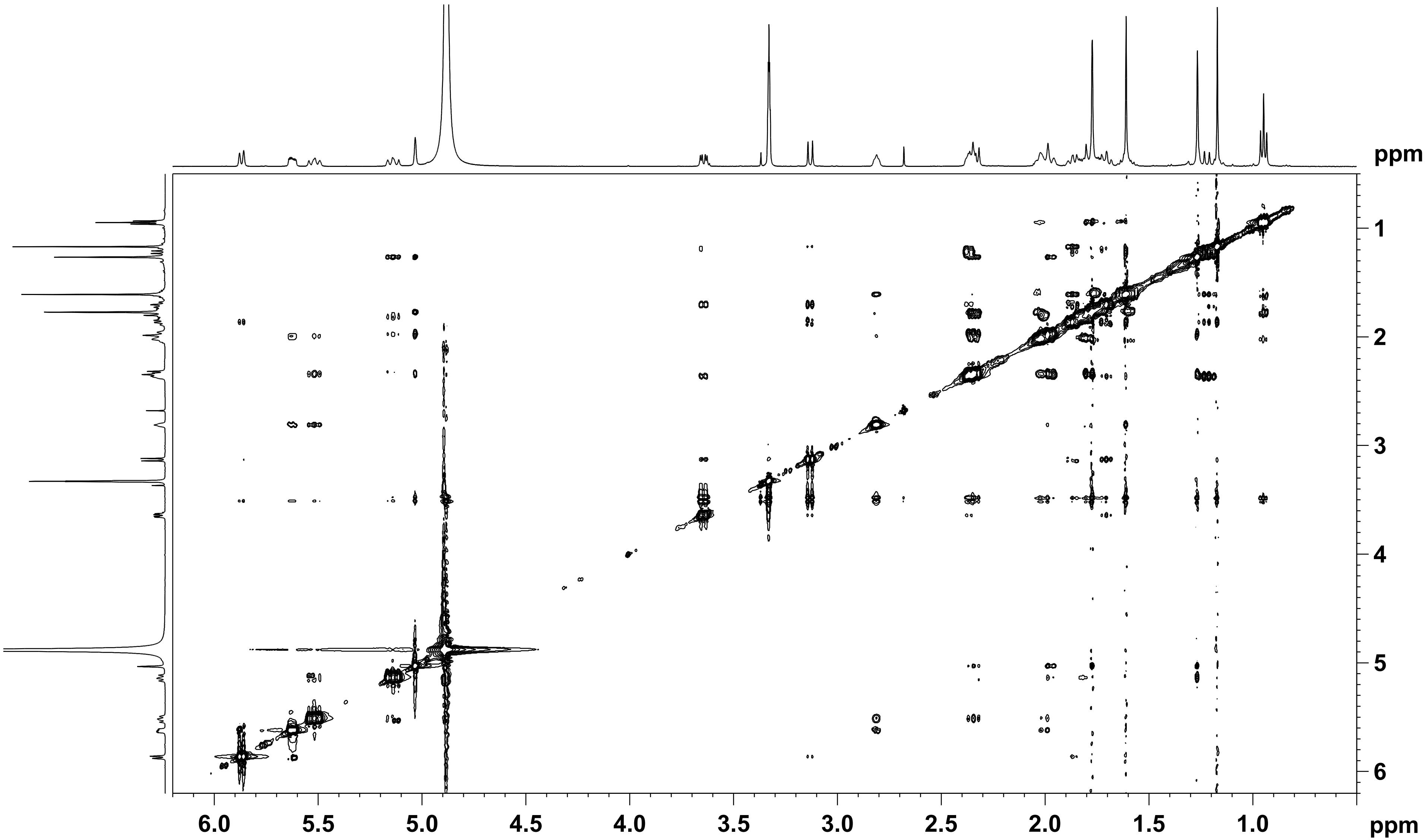

Figure S19. UV spectrum of nomimicin D (**3**).

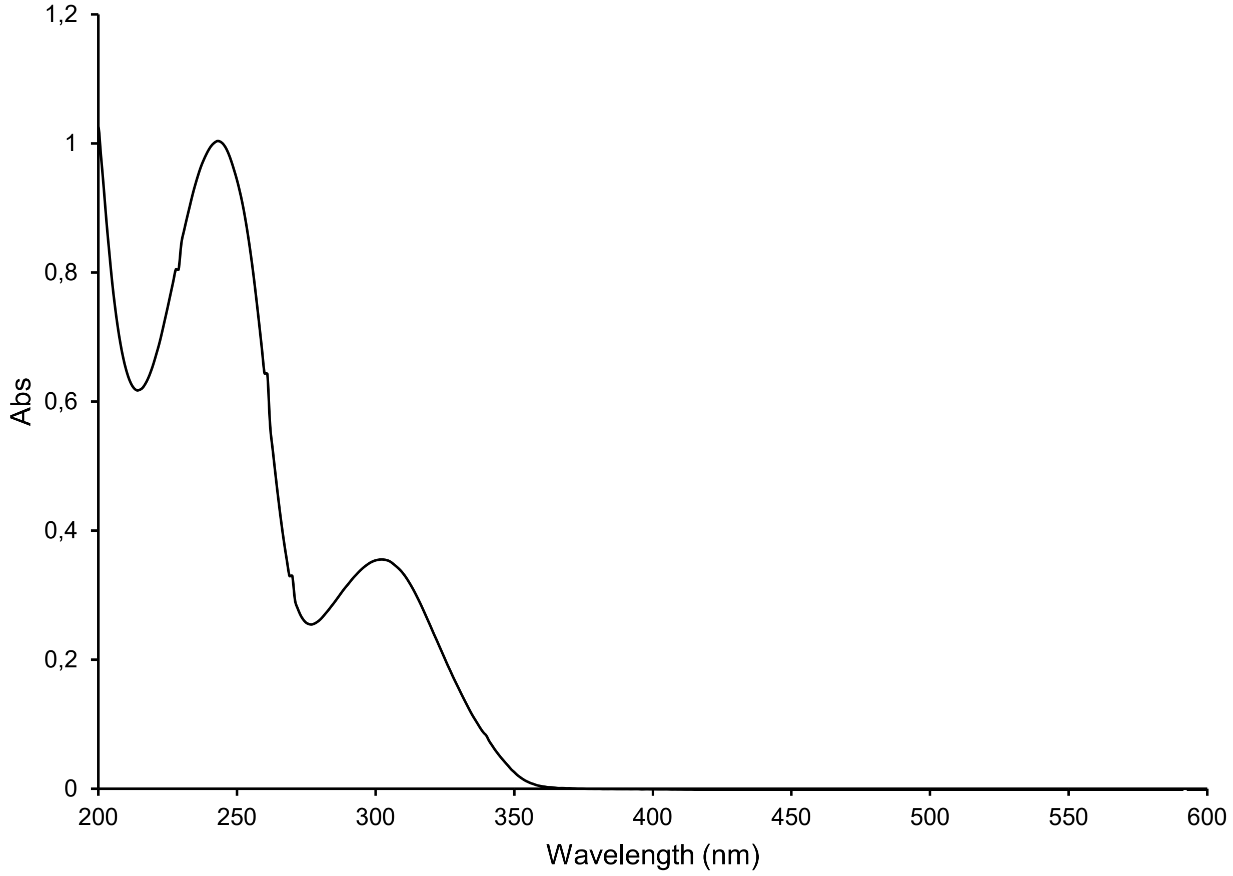

Figure S20. IR spectrum of **3.**

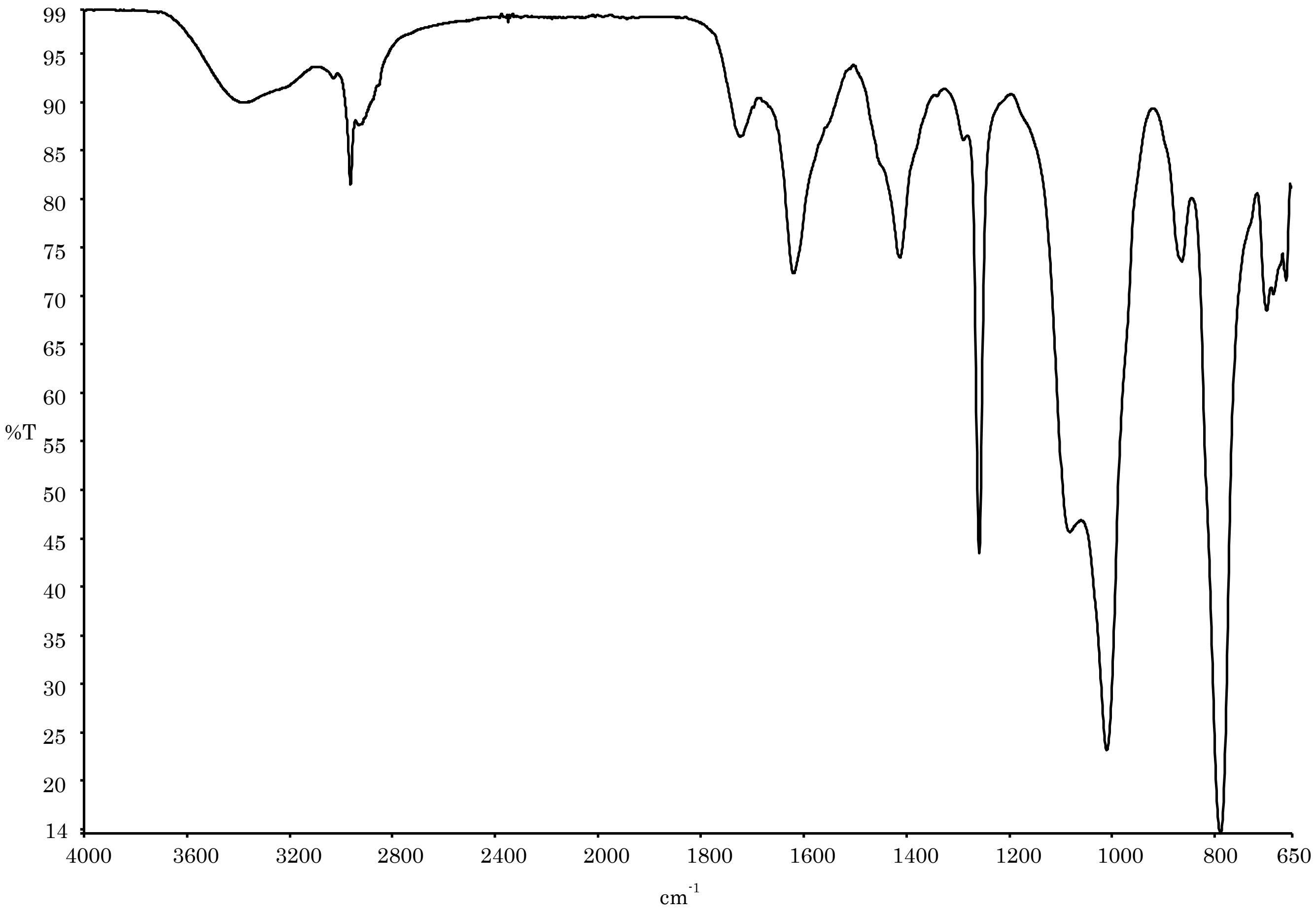

Figure S21. ^{1}H NMR spectrum of **3** (500 MHz, CD_3OD).

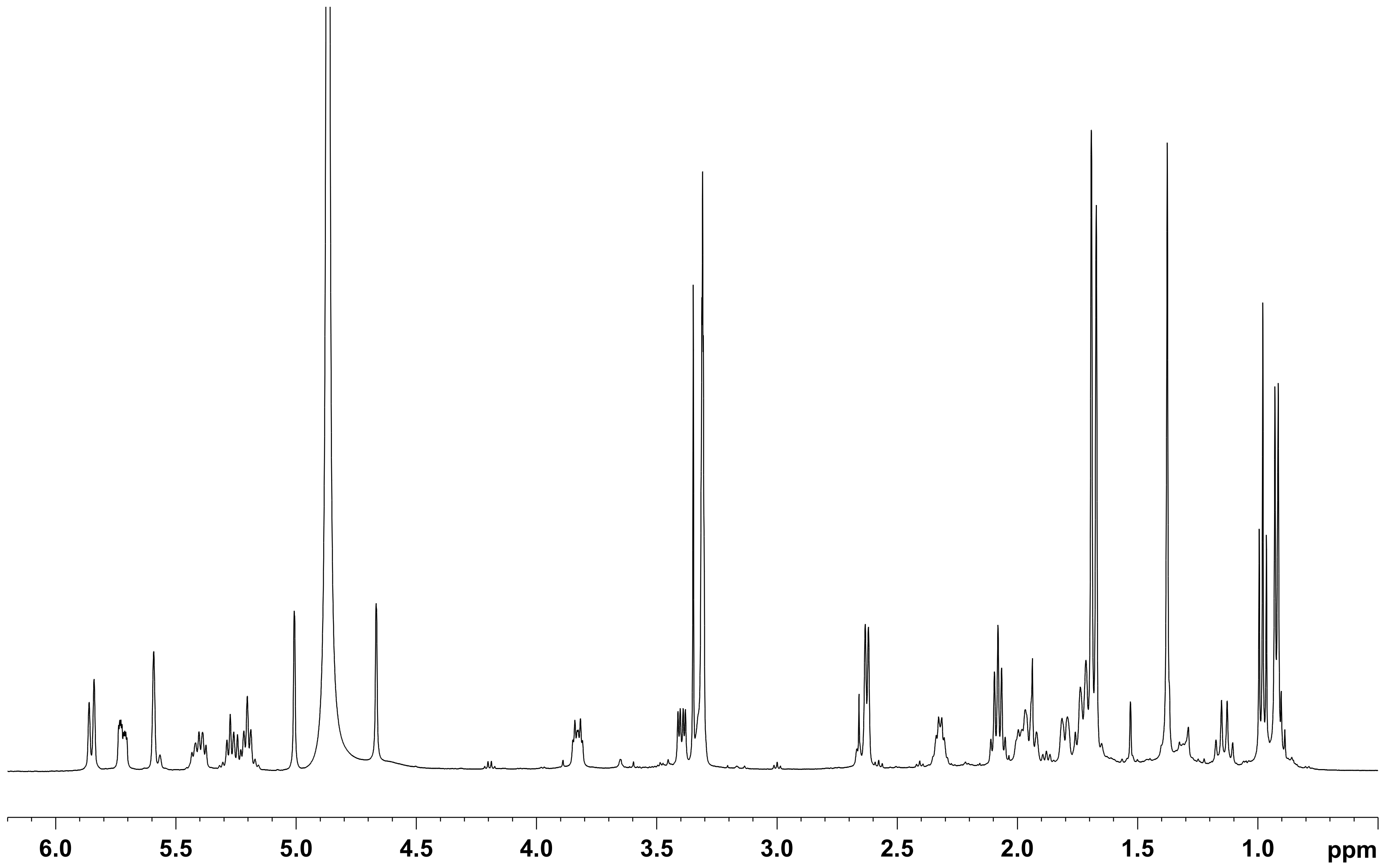

Figure S22. ^{13}C NMR spectrum of **3** (125 MHz, CD_3OD).

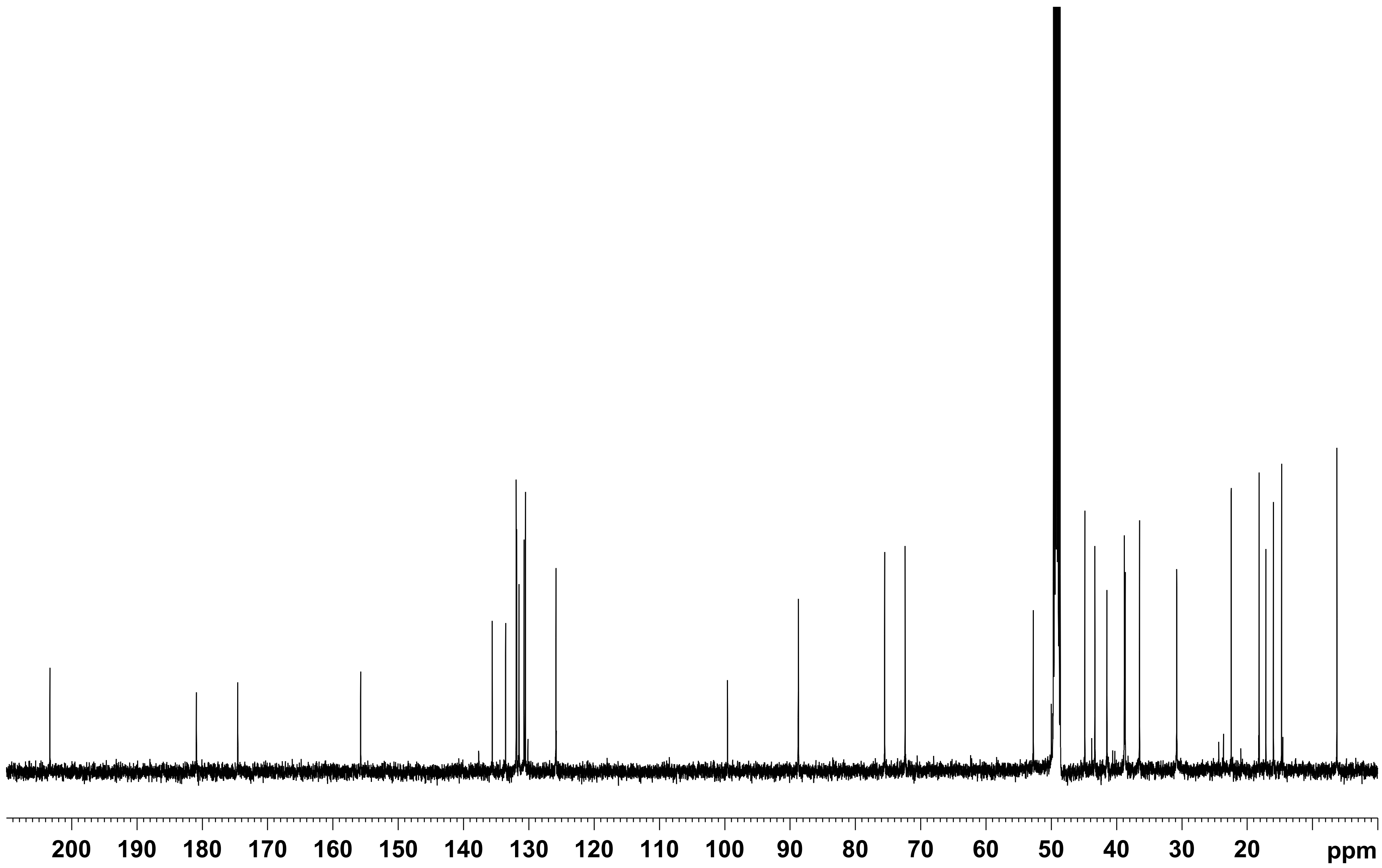

Figure S23. COSY spectrum of **3** (500 MHz, CD_3OD).

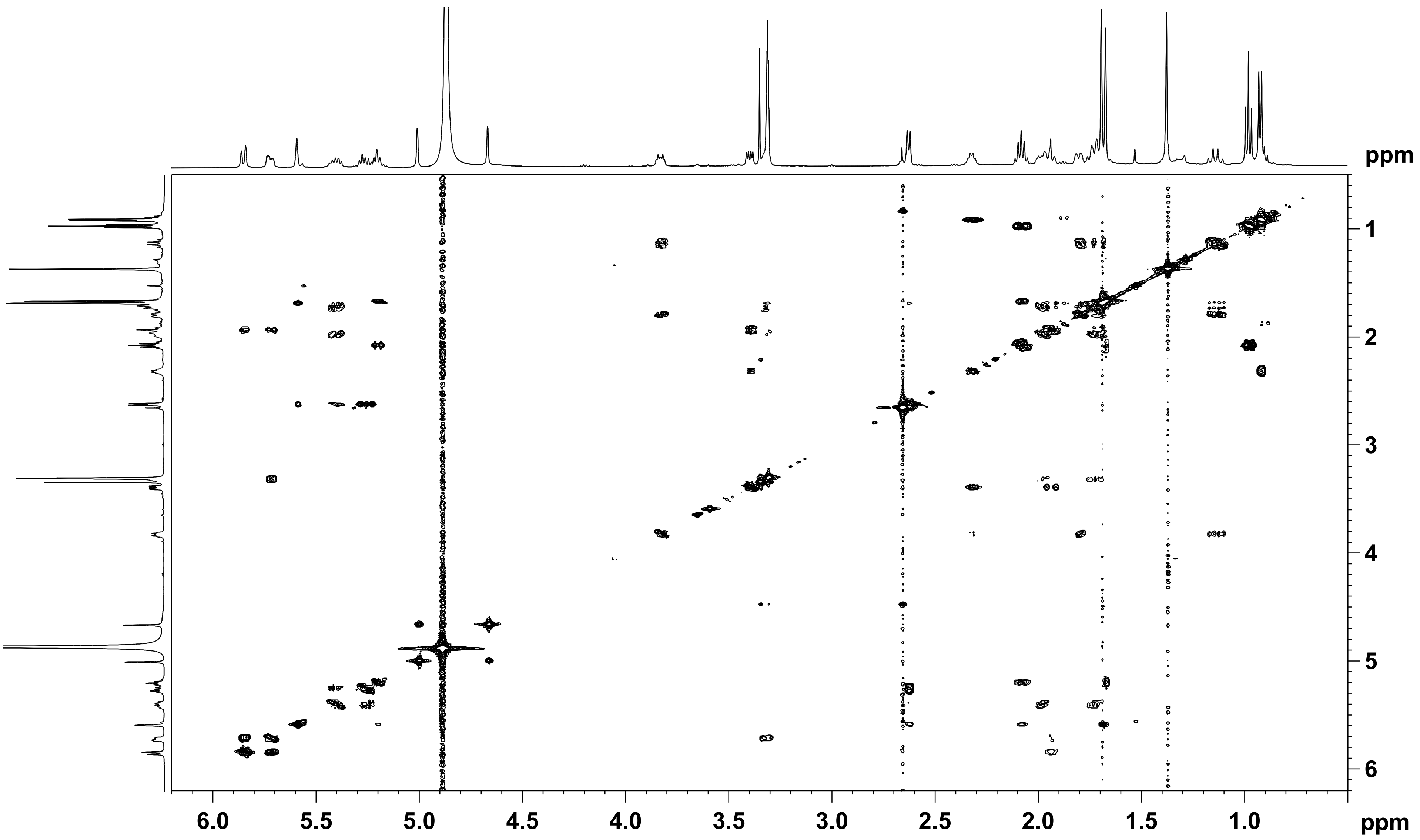

Figure S24. HSQC spectrum of **3** (500 MHz, CD_3OD).

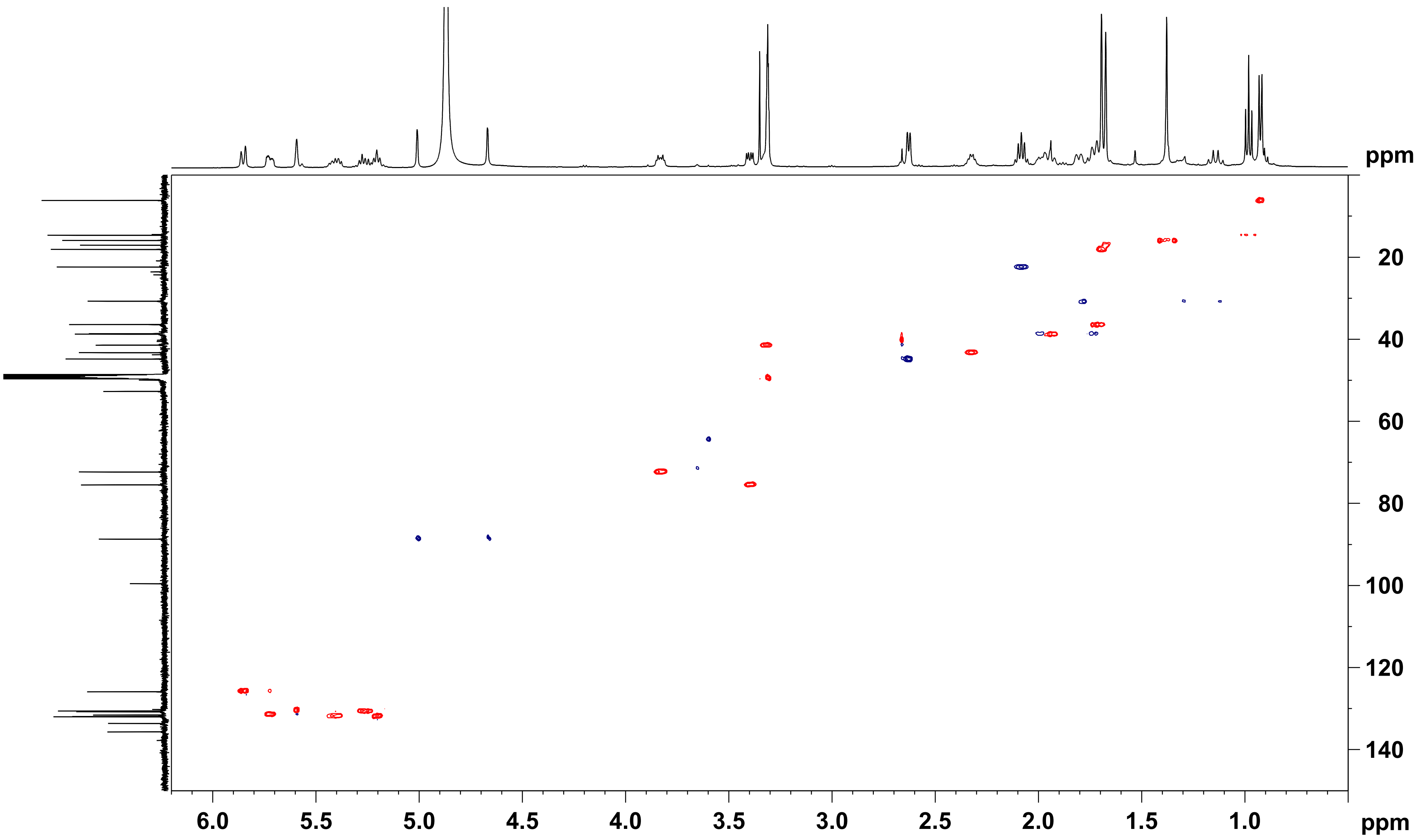

Figure S25. HMBC spectrum of **3** (500 MHz, CD_3OD).

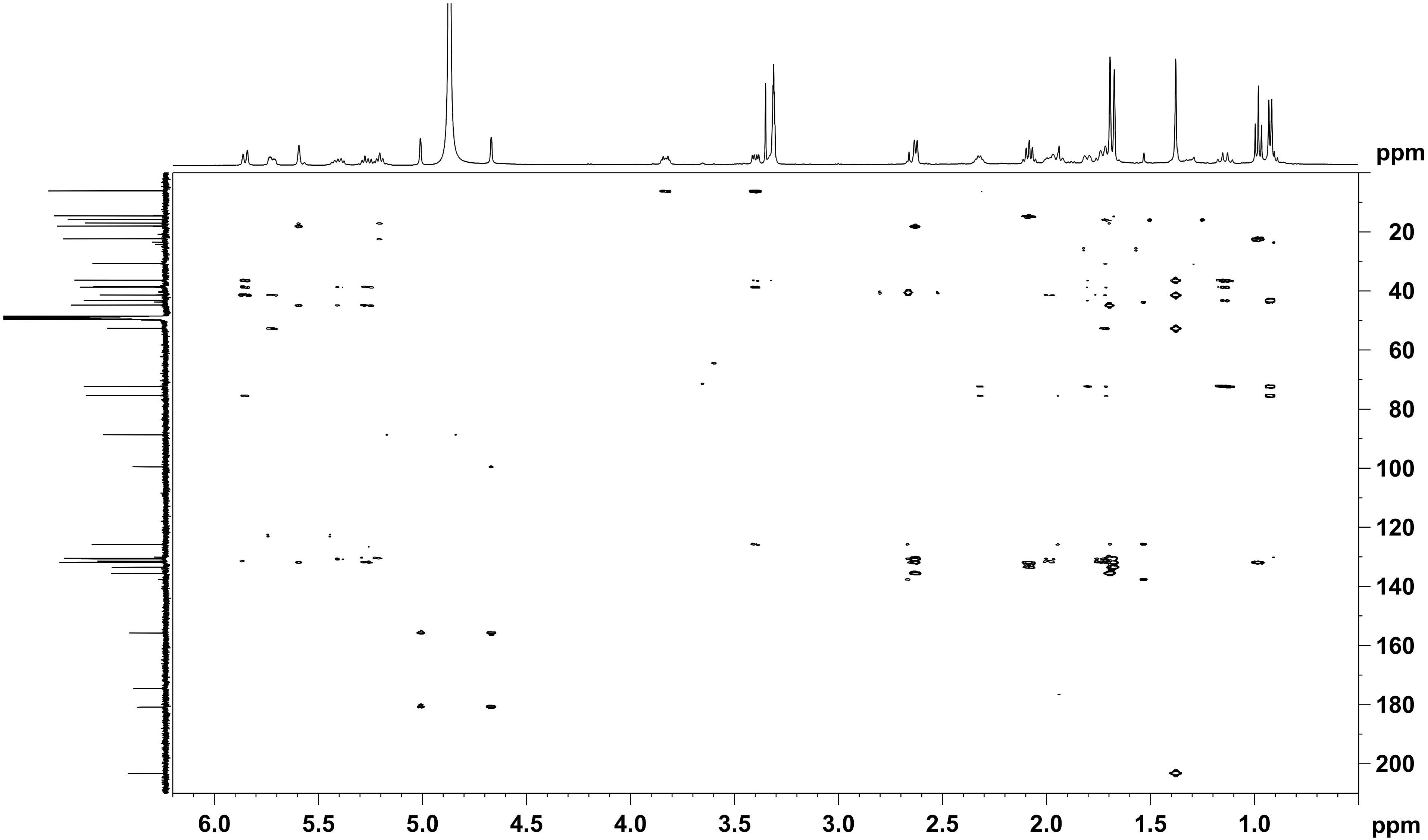

Figure S26. NOESY spectrum of **3** (500 MHz, CD_3OD).

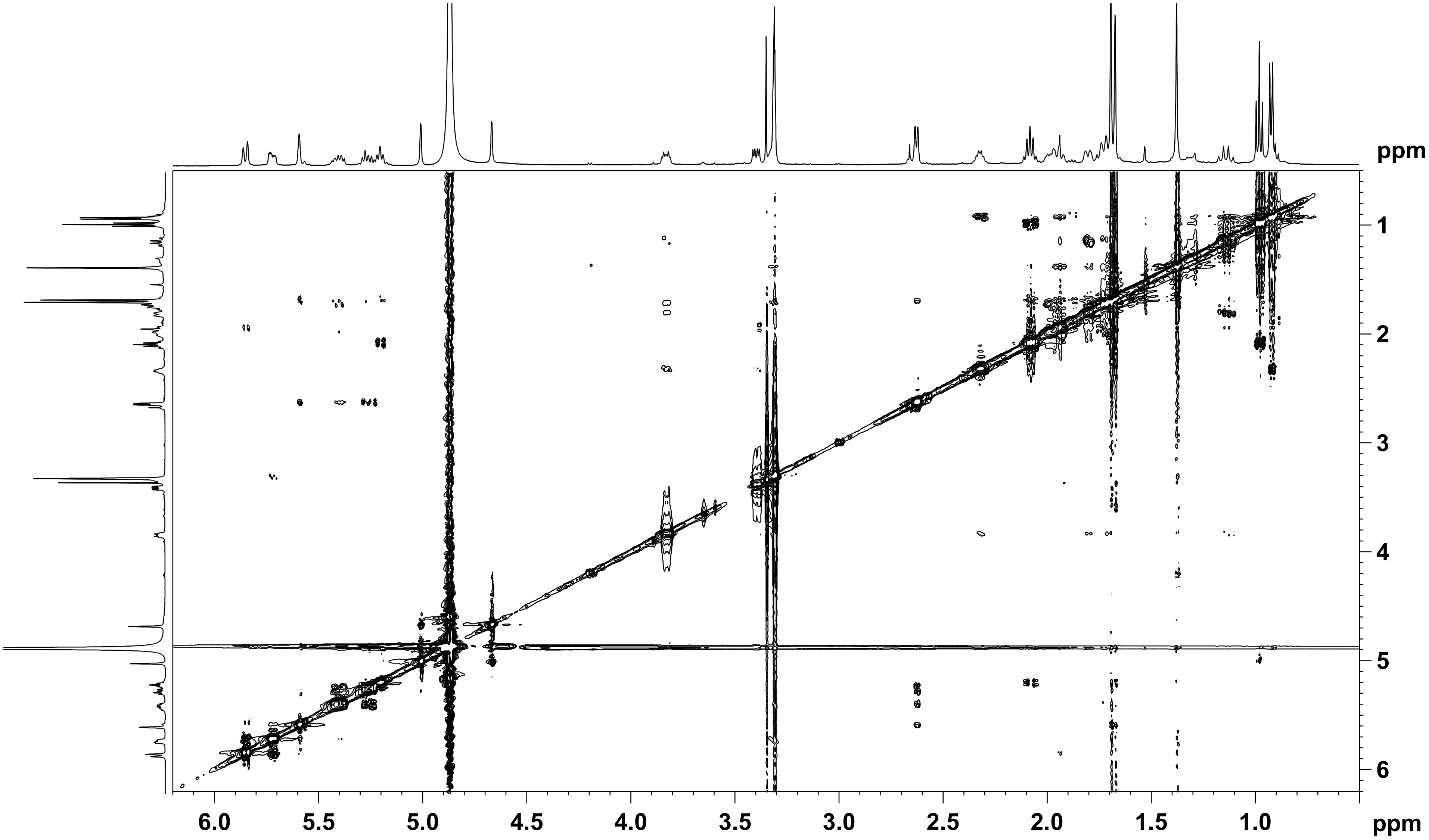

Figure S27. ROESY spectrum of **3** (500 MHz, CD_3OD).

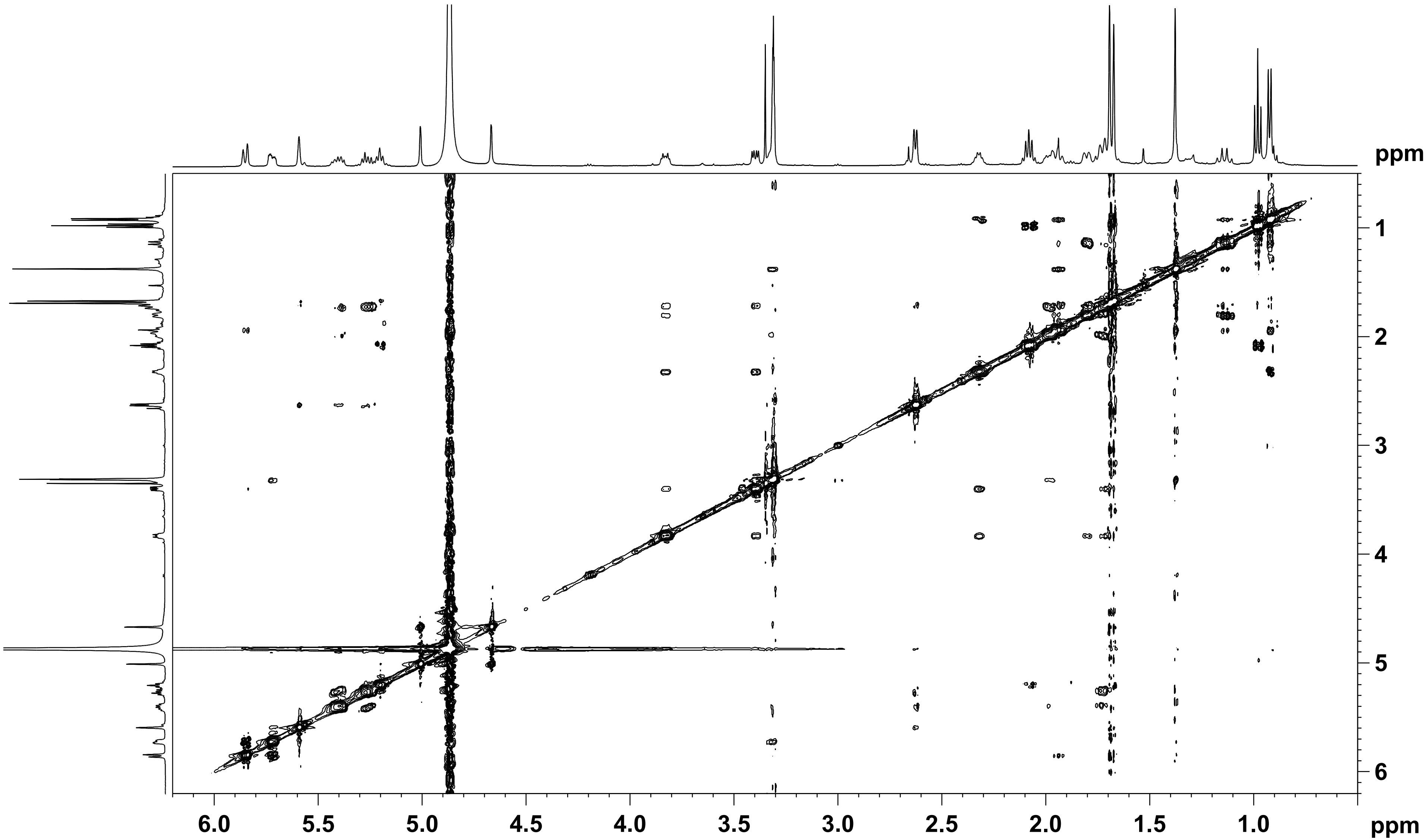

Figure S28. UV spectrum of nomimicin (**4**).

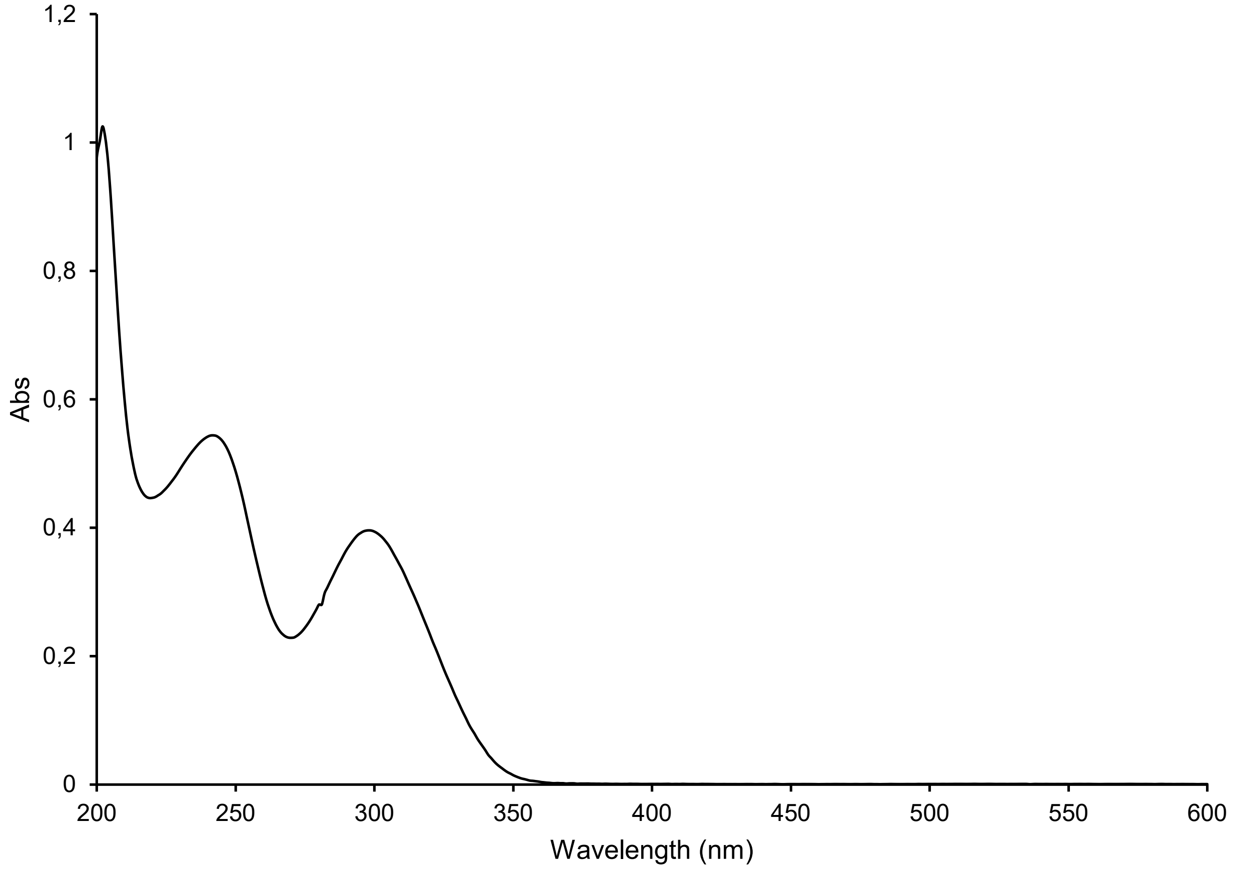

Figure S29. IR spectrum of **4.**

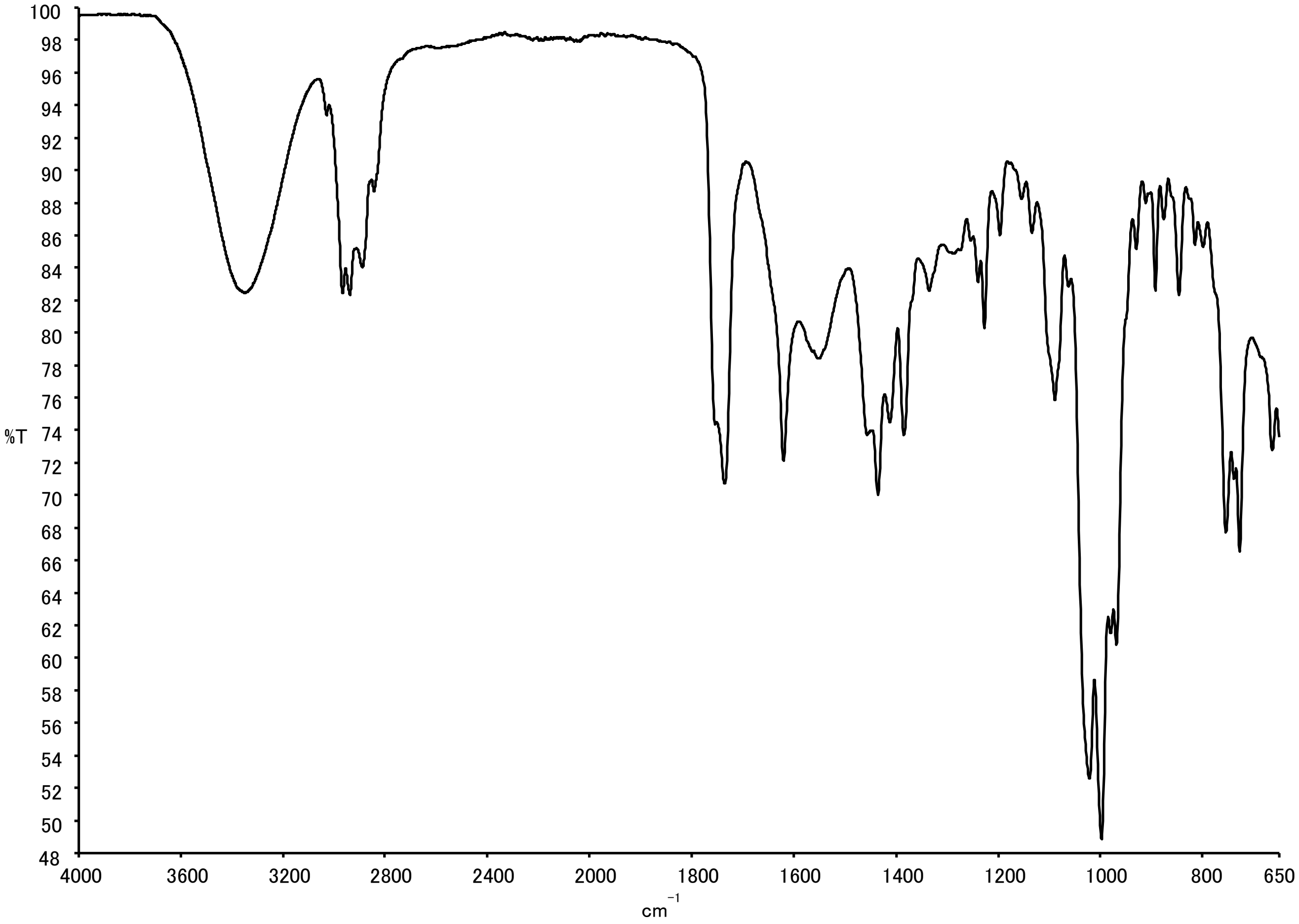

Figure S30. ^{1}H NMR spectrum of **4** (500 MHz, CD_3OD).

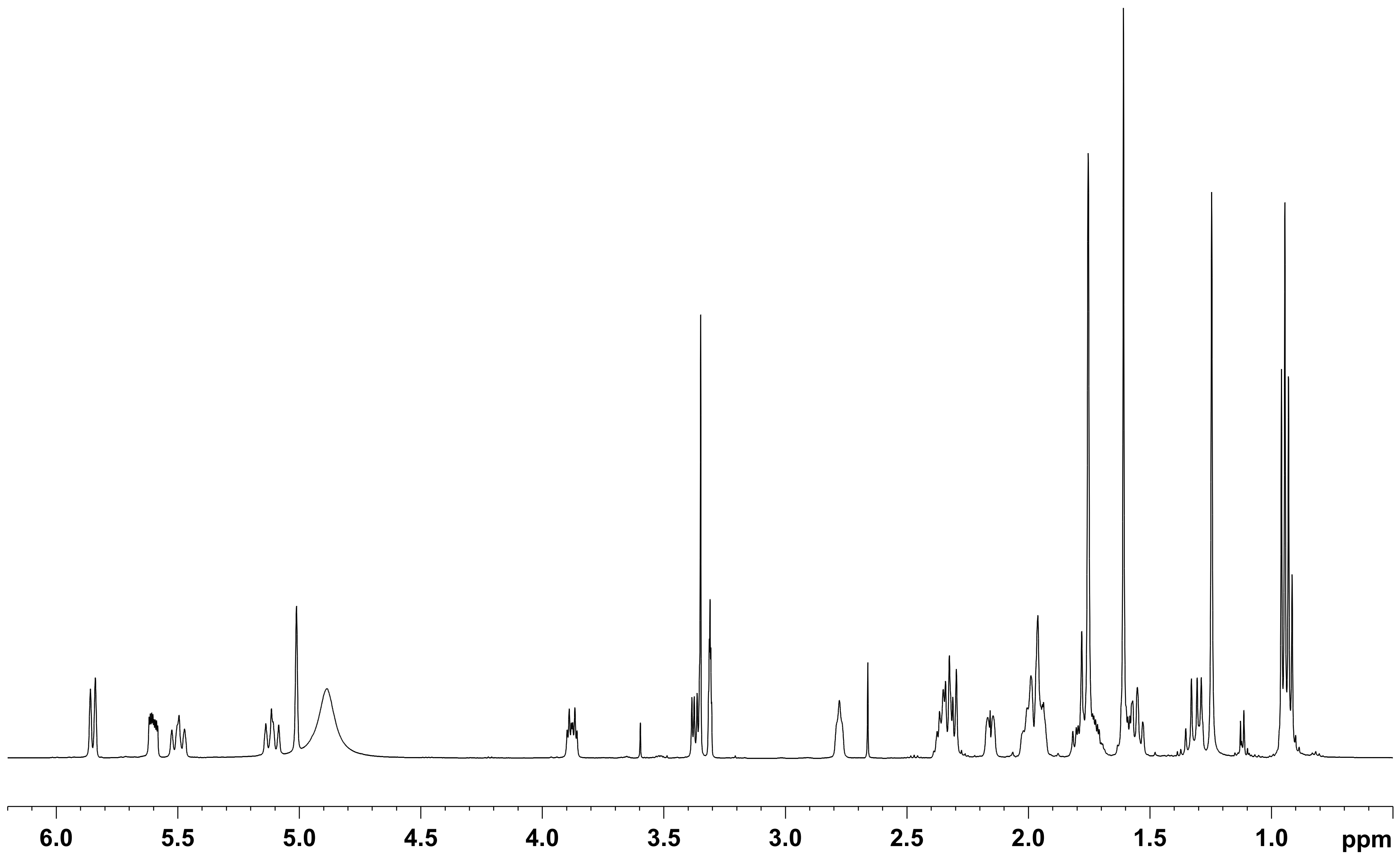

Figure S31. ^{13}C NMR spectrum of **4** (125 MHz, CD_3OD).

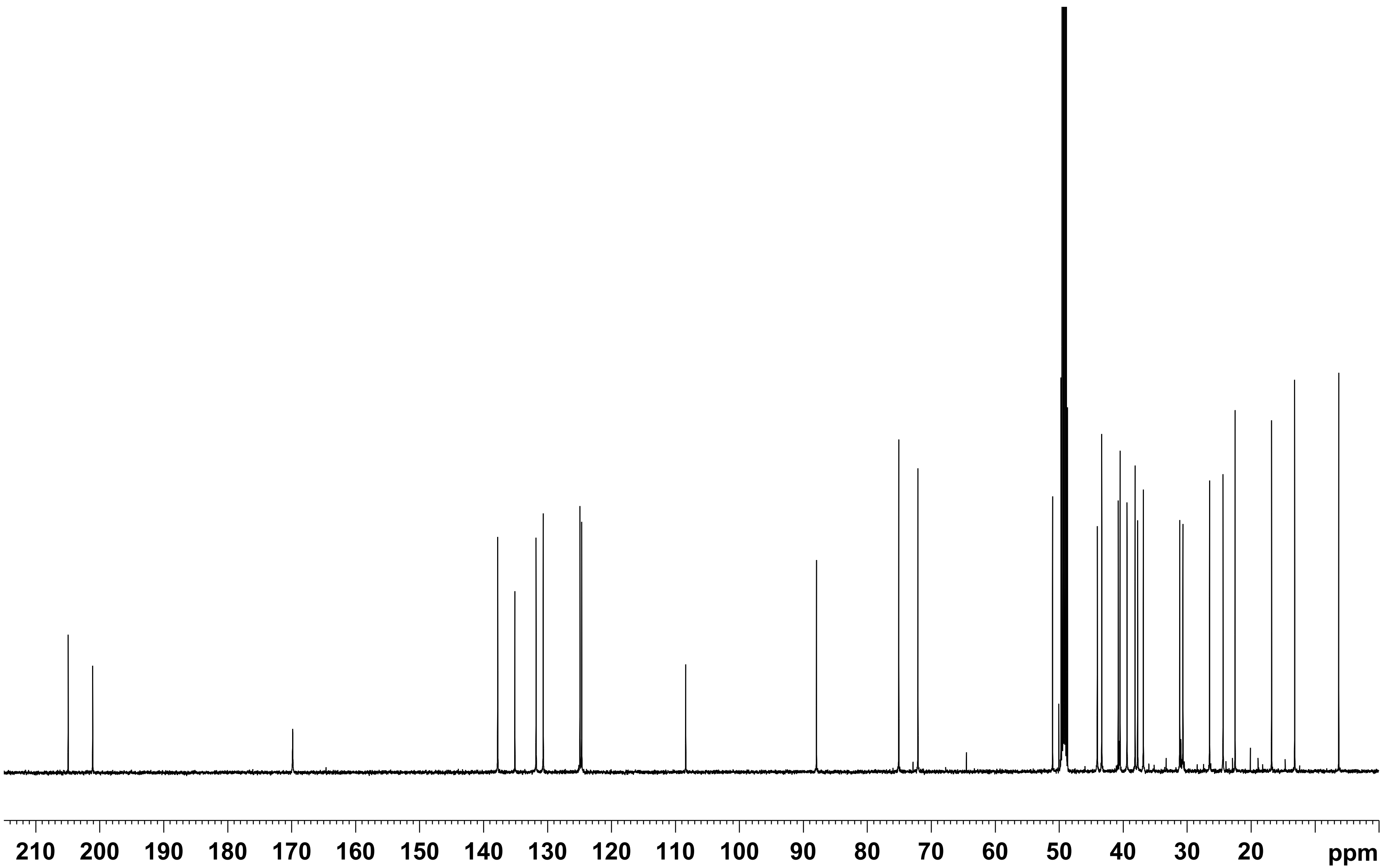

Figure S32. COSY spectrum of **4** (500 MHz, CD_3OD).

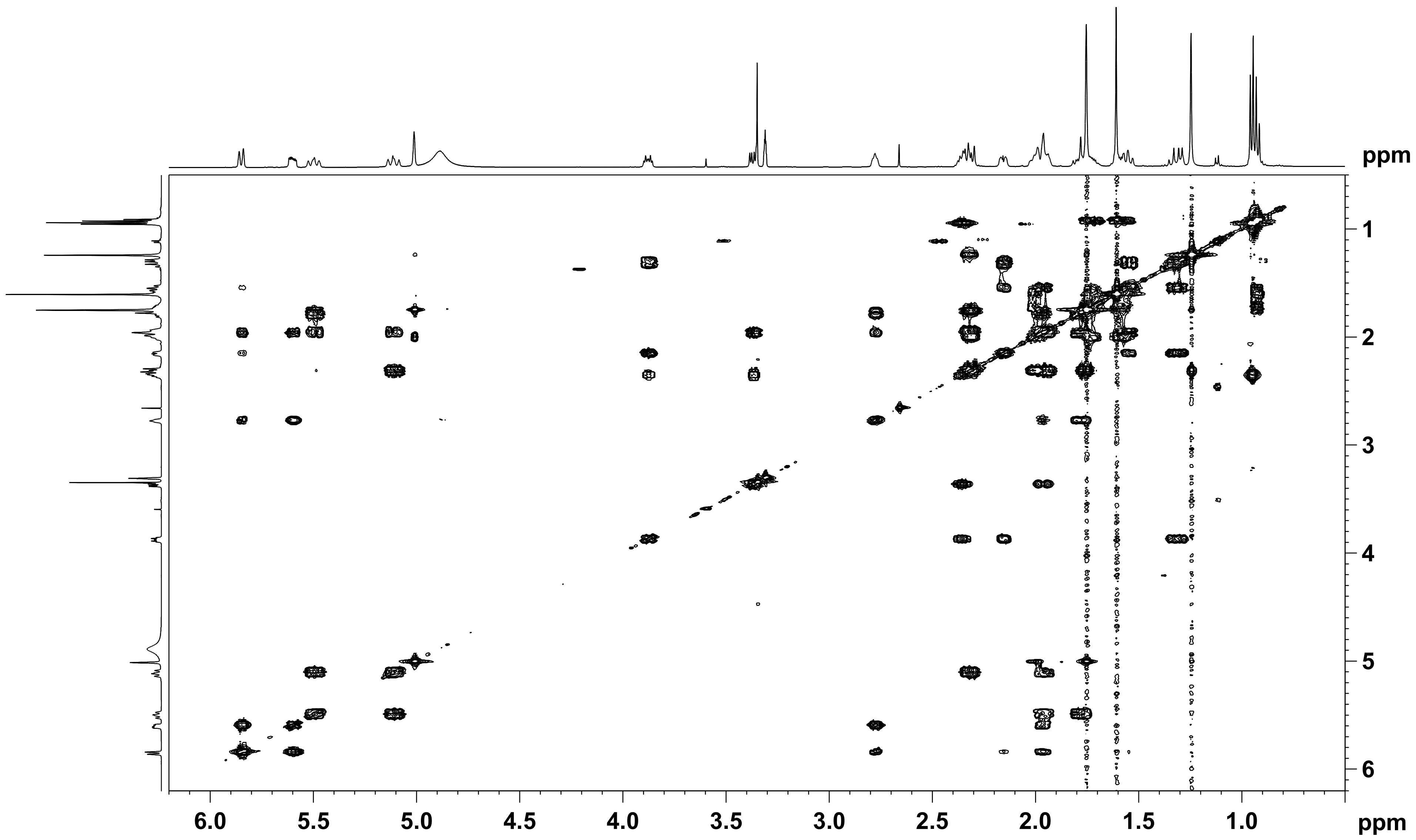

Figure S33. HSQC spectrum of **4** (500 MHz, CD_3OD).

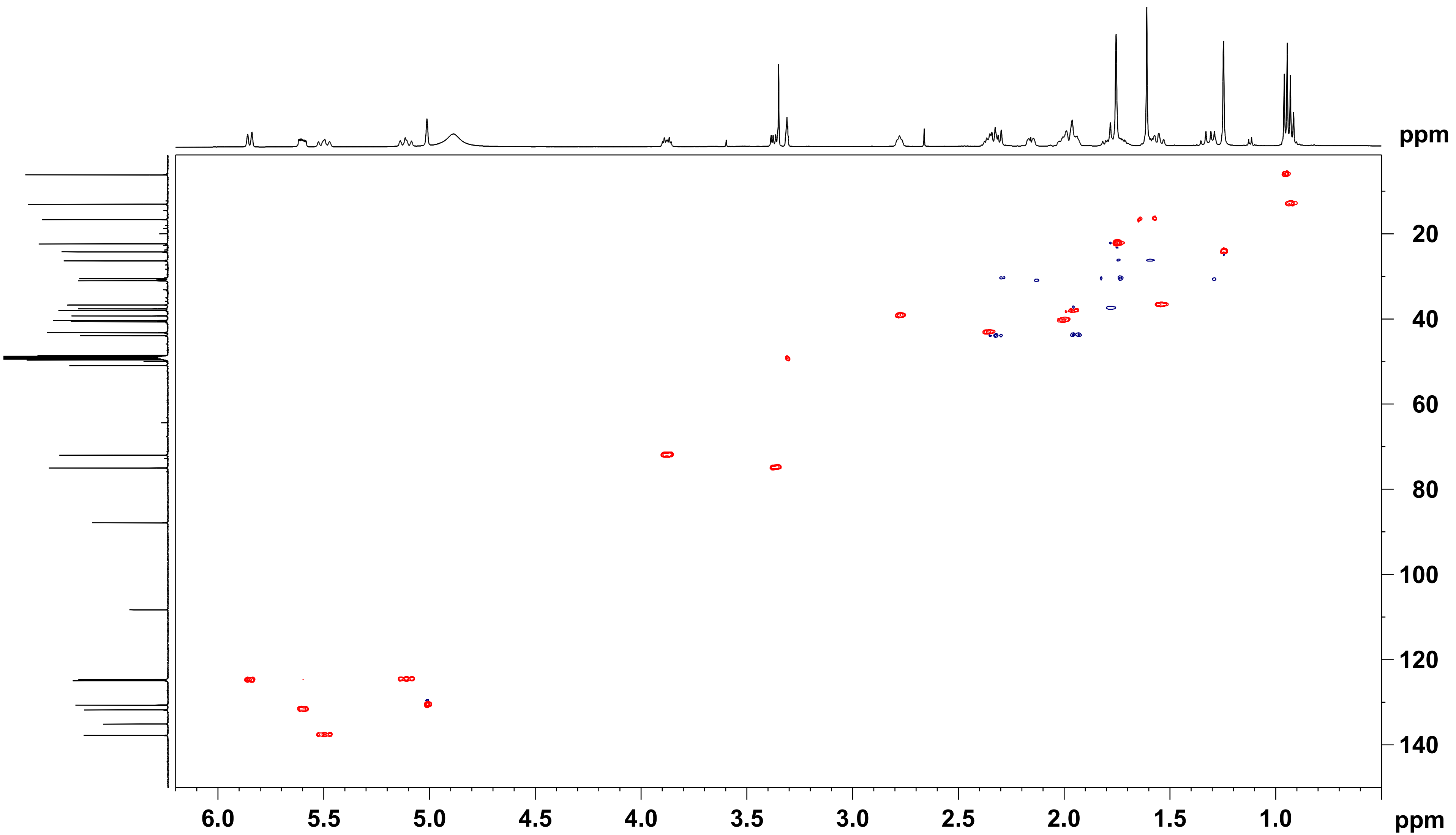

Figure S34. HMBC spectrum of **4** (500 MHz, CD_3OD).

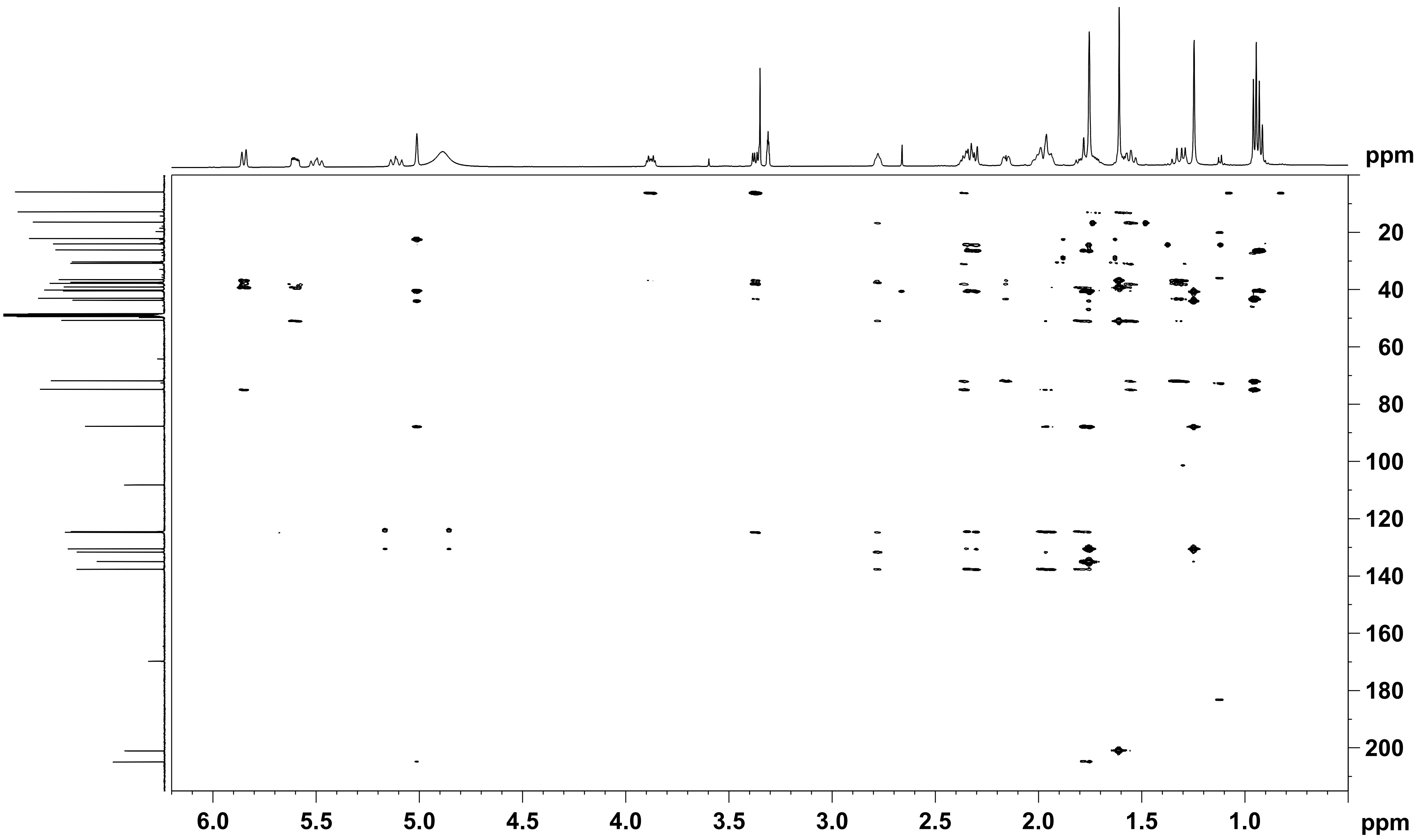

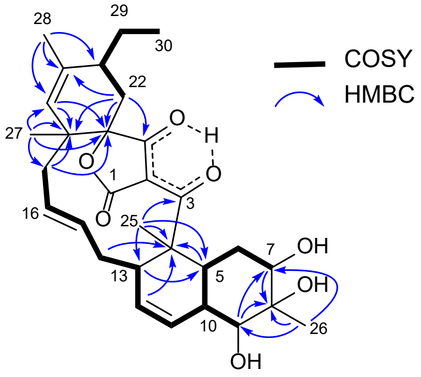

Figure S35. COSY and key HMBC correlations for **2**.

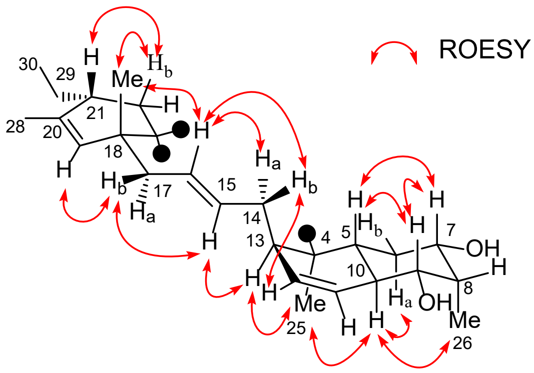

Figure S36. Relative correlations for **2** determined by ROESY analysis.

Table S1. NOESY and ROESY correlations of nomimicin B (**1**).

	nomimicin B (**1**)		
atom no.	δ_H, mult (*J* in Hz)[a]	NOESY[a]	ROESY[a]
5	1.66[b]	6b, 7, 9, 10	6b, 7, 9, 10
6ax	1.34, ddd (12.0, 12.0, 12.0)	6b, 10, 25,	6b, 10, 25, 26
6eq	2.41, brd (12.0)	5, 6a, 7	5, 6a, 7
7	3.74, dd (12.0, 4.3)	5, 6a, 9	5, 6a, 9
9	3.21, d (11.2)	5, 7	5, 7
10	2.02[b]	6a, 11, 25	6a, 11, 25, 26
11	5.85, d (10.1)	9, 10, 12	9, 10, 12
12	5.61, ddd (10.0, 5.3, 2.6)	11, 13, 14b	11, 13, 14b
13	2.81, m	12, 15, 25	12, 15, 23
14a	1.80[b]	15, 16	15, 16
14b	1.98[b]	12, 15, 16	12, 13, 15, 16
15	5.49, dd (14.7, 11.5)	13, 14a, 14b, 16, 17b	13, 14a, 14b, 16, 17b
16	5.12, dd (14.8, 11.3)	14a, 15, 17a, 17b, 27	14a, 14b, 15 17b, 27
17a	1.95[b]	27, 17b, 19	27, 17b, 19
17b	2.32[b]	15, 16, 17a, 19	17a, 19, 16, 15
19	5.00, s	17a, 17b, 27, 28	27, 28, 17a, 17b
21	2.00[b]	22b, 30	22b, 30
22a	1.78[b]	30	30
22b	2.34[b]	21, 27, 29b	21, 27, 29b
25	1.60, s	10, 13	10, 13
26	3.99, s		6b, 10
27	1.24, s		16, 17b, 19, 22b
28	1.75, s	19, 22a, 29b, 30	19
29a	1.58[d]	30	22b, 30
29b	1.72[b]	21	21, 22b
30	0.93, t (7.4)	21, 22a, 29a, 29b	21, 22a, 29a, 29b

[a]Recorded at 500 MHz. [b]Overlapping signals.

Table S2. NOESY and ROESY correlations of nomimicin C (**2**).

	nomimicin C (**2**)		
atom no.	δ_H, mult (J in Hz)[a]	NOESY[a]	ROESY[a]
5	1.68[b]	6b, 7, 9	6b, 7, 9
6ax	1.20, ddd (11.9, 11.9, 11.9)	6b, 10, 25	6b, 10, 25
6eq	2.35[b]	5, 6a, 7	5, 6a, 7
7	3.62, dd (11.8, 4.2)	5, 6a, 6b, 9	5, 6a, 6b, 9
9	3.11, d (11.0)	5, 7, 10	5, 7, 10
10	1.85[b]	6a, 9, 11, 25, 26	6a, 9, 11, 25, 26
11	5.84, d (10.0)	9, 10, 12	9, 10, 12
12	5.60, ddd (10.0, 5.1, 2.5)	11, 13, 14b	11, 13, 14b
13	2.79, m	12, 14a, 14b, 15, 25	12, 14a, 14b, 15, 25
14a	1.80[b]	13, 16	13, 16
14b	1.98[b]	12, 13, 15, 16	12, 13, 15, 16
15	5.48, dd (14.5, 11.9)	13, 14b, 16, 17b	13, 14b, 16, 17b
16	5.12, dd (14.8, 11.6)	14a, 14b, 15, 17b, 27	14a, 14b, 17b, 27
17a	1.95[b]	16, 19, 17b, 27	16, 19, 17b, 27
17b	2.32[b]	15, 19, 17a, 27	15, 16, 17b, 19, 27
19	5.01, s	17b, 21, 27, 28	17b, 21, 27, 28
21	2.01[b]	22b, 28, 30	22b, 28, 30
22a	1.79[b]	30	30
22b	2.34[b]	21, 22a, 27, 29b	21, 22a, 27, 29b
25	1.59, s	6b, 10, 13	6b, 10, 13
26	1.15, s	10	10
27	1.25, s	16, 17b, 19, 22b	16, 7b, 19, 22b
28	1.75, s	19, 21, 30	19, 21, 30
29a	1.62[b]	30	30
29b	1.75[b]	21, 22b, 30	21, 22b, 30
30	0.93, t (7.4)	21, 22a, 28, 29a, 29b	21, 22a, 28, 29a, 29b

[a]Recorded at 500 MHz. [b]Overlapping signals.

Table S3. ROESY and NOESY correlations of nomimicin D (**3**).

	nomimicin D (**3**)		
atom no.	δ_H, mult (*J* in Hz)[a]	NOESY[a]	ROESY[a]
5	1.72[b]	7	7, 9
6ax	1.14, ddd (11.7, 11.7, 11.7)	6eq, 10, 25, 26	6eq, 10, 25, 26
6eq	1.80, brd (11.7)	6ax, 7	6ax, 7
7	3.83, ddd (11.6, 4.5, 4.5)	5, 6ax, 6eq, 8	5, 6eq, 8, 9
8	2.32, m	9, 26	7, 9, 26
9	3.40, dd (10.8, 4.7)	5, 8, 10	5, 6eq, 7, 8
10	1.94[b]	6ax, 9, 11, 25, 26	6ax, 11, 26, 29
11	5.85, d (10.2)	10, 12	9, 10, 12
12	5.72, ddd (10.2, 4.8, 2.5)	11, 13	11, 13
13	3.32[b]	12, 14b, 15, 25	12, 14b, 15, 25
14a	1.75[b]	14b, 15	14b, 15, 16
14b	2.00[b]	13, 14a, 15	13, 14a, 15, 16
15	5.40, dt (15.0, 7.2)	13, 14a, 14b, 17	14a, 14b, 17
16	5.26, dt (15.2, 7.0)	17	14a, 14b, 17
17	2.63, d (6.9)	15, 16, 19, 27	15, 16, 19, 27
19	5.59, s	17, 27, 28	17, 27, 28
21	5.20, t (7.3)	28, 29	28, 29
22a	4.66, d (1.5)	22b	22b
22b	5.00, d (1.5)	22a	22a
25	1.38, s	6ax, 10, 13	6ax, 10, 13
26	0.92, d (6.9)	6ax, 8, 10	6ax, 8, 10
27	1.69, s	17, 19	17, 19
28	1.67, s	19, 21	19, 21
29	2.08, q (7.5)	21, 30	21, 30
30	0.98, t (7.5)	29	29

[a]Recorded at 500 MHz. [b]Overlapping signals.

Table S4. Cartesian coordinates and energies of the most stable conformer of **4a**.

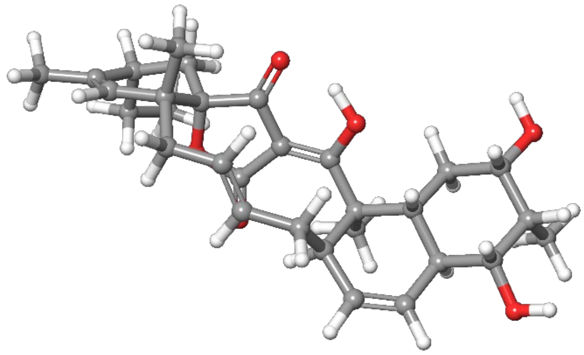

4a (ΔG = 0.0 kcal/mol)

M06-2X/def2-TZVP-SMD(MeOH)//M06-2X/6-31G(d)-SMD(MeOH):		
Gibbs Free Energy (a.u.)	=	−1617.990463
M06-2X/def2-TZVP-SMD(MeOH):		
Electronic energy (a.u.)	=	−1618.593138
M06-2X/6-31G(d)-SMD(MeOH):		
Zero-point correction (a.u.)	=	0.663531
Thermal correction to Energy (a.u.)	=	0.697126
Thermal correction to Enthalpy (a.u.)	=	0.698070
Thermal correction to Gibbs Free Energy (a.u.)	=	0.602675

C	−3.503719	−2.346714	2.018768	C	−3.645152	−0.928348	2.483653
C	−2.842684	0.059614	1.614795	C	−1.406663	−0.467423	1.327473
C	−1.478556	−1.858223	0.592342	C	−2.535046	−2.744197	1.197996
C	−5.114423	−0.482563	2.517646	C	−5.279711	0.929994	3.107325
C	−4.385326	1.900074	2.329127	C	−2.928249	1.436913	2.295338
H	−3.357511	0.126062	0.644467	H	−3.275366	−0.872508	3.519517
C	−5.025003	0.971896	4.615534	O	−5.838844	−1.443966	3.275191
O	−4.511032	3.183884	2.931226	C	−0.628314	−0.610233	2.647818
C	−0.659504	0.477094	0.398921	C	0.661656	0.373915	−0.030811
O	−1.393789	1.424828	−0.127383	C	1.639477	−0.706686	0.113186
O	2.586816	−0.577666	−0.849731	C	2.365451	0.552627	−1.719717
C	1.091115	1.157639	−1.160964	O	0.536366	2.160395	−1.624592
C	3.524265	1.533137	−1.535198	O	1.725959	−1.631641	0.893988
C	−1.689680	−1.808606	−0.957891	C	−0.406444	−1.965046	−1.729979
C	0.043227	−1.135931	−2.672441	C	1.404868	−1.243425	−3.302139
C	2.266578	0.047683	−3.188329	C	1.683546	1.118875	−4.130266

C	3.662248	−0.283699	−3.677645
C	4.887106	0.905325	−1.852568
C	5.512444	0.066671	−0.711618
H	−4.220916	−3.069641	2.399844
H	−2.480596	−3.789269	0.893961
H	−6.322708	1.233321	2.937115
H	−2.550962	1.377486	3.322789
H	−3.989715	0.728106	4.874078
H	−5.672929	0.256624	5.129063
H	−3.988011	3.806797	2.401920
H	0.400098	−0.923285	2.472441
H	−0.845789	1.972098	−0.768030
H	3.499613	1.926940	−0.514148
H	−2.207474	−0.885844	−1.250439
H	−0.577815	−0.283741	−2.958990
H	1.318885	−1.457654	−4.376482
H	0.619692	1.297559	−3.956449
H	3.681631	−0.872176	−4.596844
H	5.999076	−0.804052	−4.700691
H	6.676972	−1.020392	−3.079586
H	6.565455	−0.099793	−0.966336
H	5.792408	1.755548	0.637118

C	4.821642	0.087611	−3.125828
C	6.139258	−0.320159	−3.729921
C	5.437464	0.718326	0.666353
H	−0.513522	−2.347953	0.765753
H	−5.489581	−0.477409	1.481599
H	−4.751292	1.947582	1.290979
H	−2.335827	2.192927	1.774845
H	−5.241348	1.967971	5.010081
H	−6.770566	−1.171176	3.284207
H	−1.108293	−1.368329	3.274221
H	−0.614276	0.335119	3.197890
H	3.343562	2.379927	−2.204942
H	−2.362498	−2.633450	−1.225418
H	0.219027	−2.810432	−1.431182
H	1.944444	−2.082350	−2.849352
H	2.210249	2.072954	−4.037394
H	1.805293	0.771193	−5.161667
H	5.577214	1.743265	−2.030130
H	6.791306	0.551283	−3.866927
H	5.039526	−0.920376	−0.677361
H	6.060169	0.172370	1.382180
H	4.415026	0.722727	1.058638

Table S5. Cartesian coordinates and energies of the most stable conformer of **4b**.

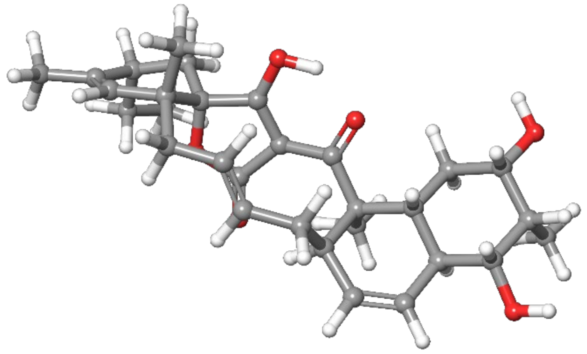

4b (Δ*G* = 0.4 kcal/mol)

M06-2X/def2-TZVP-SMD(MeOH)//M06-2X/6-31G(d)-SMD(MeOH):			
	Gibbs Free Energy (a.u.)	=	−1617.989748
M06-2X/def2-TZVP-SMD(MeOH):			
	Electronic energy (a.u.)	=	−1618.592155
M06-2X/6-31G(d)-SMD(MeOH):			
	Zero-point correction (a.u.)	=	0.663404
	Thermal correction to Energy (a.u.)	=	0.697073
	Thermal correction to Enthalpy (a.u.)	=	0.698017
	Thermal correction to Gibbs Free Energy (a.u.)	=	0.602407

C	3.618438	−2.909456	−0.579758
C	2.246516	−2.007607	1.341583
C	1.574001	−1.515065	−1.078718
C	3.919407	−3.832798	1.738119
C	2.724025	−2.801589	3.672159
H	1.506189	−2.821987	1.306460
C	5.268592	−2.681520	3.576455
O	2.861065	−2.450254	5.045375
C	0.375484	−0.398696	0.810911
O	−0.219862	−0.956279	1.743585
O	−1.298307	1.950198	−1.549630
C	−1.691651	0.841546	0.399706
C	−2.894037	2.981673	−0.067781
C	0.236993	−2.284458	−1.351032
C	−2.006901	−1.178225	−1.698225
C	−3.499885	0.907016	−1.437439
C	3.596050	−2.596963	0.886760
C	1.744913	−0.909585	0.363405
C	2.729540	−2.413072	−1.436418
C	4.018140	−3.495126	3.236562
C	2.405109	−1.579392	2.810437
H	4.389366	−1.859668	1.088091
O	5.138024	−4.385086	1.254332
C	2.738885	0.264248	0.324390
C	−0.358178	0.669337	0.118117
C	−0.133183	1.376208	−1.147617
C	−2.383160	1.661905	−0.649570
O	−2.367565	0.368997	1.407338
O	0.855282	1.518871	−1.836063
C	−0.766837	−1.451250	−2.103366
C	−2.919243	−0.192170	−2.376254
C	−4.542970	0.261648	−0.504280

C	−4.225278	1.915965	−2.305660
C	−3.363629	3.958870	−1.152301
C	−2.242858	4.779816	−1.835195
H	4.416873	−3.553661	−0.940213
H	2.794035	−2.675314	−2.492315
H	4.082399	−4.448033	3.781395
H	3.219561	−0.852265	2.910908
H	5.263195	−1.689580	3.113726
H	6.165481	−3.203811	3.232878
H	2.022055	−2.053943	5.329490
H	2.368992	1.081147	−0.295383
H	−1.734714	−0.221670	1.908174
H	−2.106467	3.424195	0.549511
H	−0.195437	−2.653647	−0.411959
H	−2.362485	−1.633739	−0.770359
H	−3.789946	−0.705525	−2.807762
H	−4.154491	−0.614174	0.020779
H	−4.847764	1.463781	−3.079838
H	−5.641434	3.529251	−3.777741
H	−4.314530	4.701212	−3.791520
H	−2.723787	5.600685	−2.379436
H	−1.694869	5.890488	−0.041819

C	−4.198722	3.247720	−2.196414
C	−4.980385	4.122875	−3.139989
C	−1.211742	5.364777	−0.874187
H	1.602124	−0.670138	−1.775574
H	3.106877	−4.563318	1.595092
H	1.896101	−3.520285	3.561892
H	1.498088	−1.110394	3.197653
H	5.350434	−2.544297	4.657764
H	5.332275	−5.177042	1.781278
H	3.690265	−0.070811	−0.101061
H	2.929024	0.652284	1.329640
H	−3.727638	2.745672	0.601172
H	0.479290	−3.173280	−1.947303
H	−0.396145	−0.983866	−3.019317
H	−2.383595	0.282214	−3.205743
H	−4.919434	0.969573	0.239730
H	−5.393040	−0.060747	−1.114758
H	−4.013156	4.688077	−0.646375
H	−5.590130	4.846452	−2.585113
H	−1.737548	4.161791	−2.584728
H	−0.568568	6.082041	−1.393859
H	−0.563519	4.589250	−0.452792

Table S6. Cartesian coordinates and energies of the most stable conformer of **4c**.

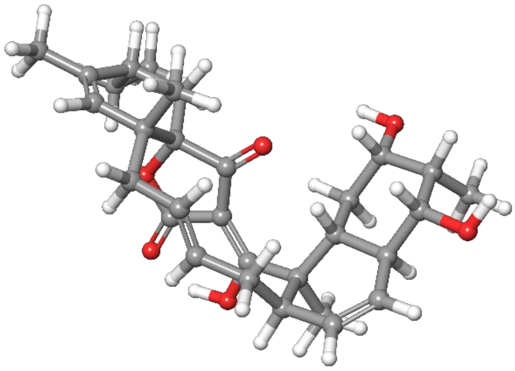

4c (ΔG = 3.5 kcal/mol)

M06-2X/def2-TZVP-SMD(MeOH)//M06-2X/6-31G(d)-SMD(MeOH):		
Gibbs Free Energy (a.u.)	=	−1617.984871
M06-2X/def2-TZVP-SMD(MeOH):		
Electronic energy (a.u.)	=	−1618.586628
M06-2X/6-31G(d)-SMD(MeOH):		
Zero-point correction (a.u.)	=	0.663069
Thermal correction to Energy (a.u.)	=	0.696797
Thermal correction to Enthalpy (a.u.)	=	0.697741
Thermal correction to Gibbs Free Energy (a.u.)	=	0.601757

C	1.860459	−3.459310	−2.831985
C	1.197889	−1.132826	−2.052228
C	−0.397974	−3.086560	−1.796580
C	3.612081	−1.676168	−2.374454
C	2.903567	0.685037	−1.919253
H	1.415731	−1.295168	−0.991161
C	4.147735	0.154664	−4.080021
O	3.221913	2.045455	−2.197232
C	−1.344627	−0.781702	−1.620529
O	−2.502099	−0.999269	−2.203615
O	−2.496049	0.957098	1.285166
C	−0.347569	0.355626	0.533365
C	−0.695930	2.371282	2.000210
C	−0.295529	−3.391619	−0.274381
C	−0.820582	−2.195345	1.841232
C	−1.054727	0.061423	3.016344
C	2.192833	−1.993084	−2.859413
C	−0.251763	−1.602591	−2.299214
C	0.682014	−3.934794	−2.429161
C	3.961273	−0.191477	−2.601203
C	1.480742	0.340058	−2.363188
H	2.161243	−1.680349	−3.914794
O	4.512152	−2.530782	−3.068664
C	−0.570394	−1.573346	−3.814119
C	−1.334381	0.041244	−0.494802
C	−2.617691	0.454941	0.056409
C	−1.117345	0.929387	1.731912
O	0.868793	0.270088	0.544045
O	−3.731874	0.363810	−0.458461
C	−1.229319	−2.685563	0.669919
C	−1.643751	−1.358336	2.779778
C	0.384052	−0.061543	3.556985

C	−1.876495	0.775743	4.069110
C	−1.571302	3.060906	3.068040
C	−2.699898	3.912004	2.446892
H	2.611150	−4.140192	−3.226940
H	0.488758	−5.006254	−2.460316
H	4.918040	−0.003764	−2.092933
H	1.373687	0.533681	−3.438007
H	3.214072	0.091985	−4.647964
H	4.867138	−0.526239	−4.542819
H	2.599991	2.595456	−1.694684
H	−1.522805	−2.061216	−4.026022
H	−3.234325	−0.509748	−1.722967
H	−0.745179	2.919170	1.052934
H	0.735084	−3.218691	0.057057
H	0.222227	−2.350860	2.121294
H	−1.692504	−1.838057	3.766809
H	1.077931	−0.486199	2.829223
H	−2.256280	0.143831	4.873184
H	−3.013864	1.981457	6.063707
H	−3.902115	2.996720	4.921281
H	−3.336836	4.291939	3.252575
H	−1.556937	5.749962	2.267190

C	−2.106078	2.091865	4.110946
C	−2.898741	2.695141	5.243048
C	−2.190515	5.107404	1.644890
H	−1.381654	−3.435593	−2.136929
H	3.665526	−1.890851	−1.295217
H	2.965445	0.510769	−0.835070
H	0.781958	1.002806	−1.841389
H	4.531124	1.173008	−4.184646
H	5.409371	−2.331953	−2.755270
H	0.201758	−2.095590	−4.377998
H	−0.627951	−0.542631	−4.178704
H	0.356037	2.356798	2.297764
H	−0.457608	−4.476815	−0.198116
H	−2.268453	−2.554477	0.356545
H	−2.673820	−1.276961	2.412699
H	0.769758	0.911027	3.873369
H	0.368726	−0.711604	4.439169
H	−0.924534	3.766509	3.612048
H	−2.404578	3.593578	5.632692
H	−3.333373	3.274692	1.818785
H	−3.027840	5.713157	1.283832
H	−1.604970	4.807807	0.770002

Table S7. Cartesian coordinates and energies of the most stable conformer of **4d**.

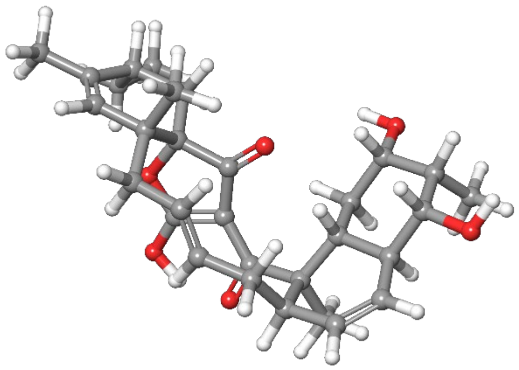

4d (ΔG = 6.6 kcal/mol)

M06-2X/def2-TZVP-SMD(MeOH)//M06-2X/6-31G(d)-SMD(MeOH):		
Gibbs Free Energy (a.u.)	=	−1617.979974
M06-2X/def2-TZVP-SMD(MeOH):		
Electronic energy (a.u.)	=	−1618.580947
M06-2X/6-31G(d)-SMD(MeOH):		
Zero-point correction (a.u.)	=	0.662219
Thermal correction to Energy (a.u.)	=	0.695993
Thermal correction to Enthalpy (a.u.)	=	0.696937
Thermal correction to Gibbs Free Energy (a.u.)	=	0.600973

C	4.798129	0.356612	−0.661757
C	2.478207	−0.672378	−0.665771
C	3.296870	0.647454	1.332342
C	3.674606	−0.330715	−2.832130
C	1.516062	−1.602040	−2.775862
H	1.948008	0.249241	−0.931338
C	3.620670	−2.762442	−3.625970
O	0.804306	−2.670629	−3.393564
C	1.320249	−0.869455	1.671112
O	1.468333	−1.303555	2.834544
O	−2.154086	0.042689	2.009603
C	−0.683430	0.158578	0.171270
C	−3.177513	−0.099993	−0.157614
C	2.526872	1.990994	1.182403
C	0.151547	2.728125	1.218604
C	−2.290452	2.111794	0.754683
C	3.865655	−0.611239	−1.337749
C	2.621313	−0.693659	0.870177
C	4.576705	0.857650	0.553923
C	2.852422	−1.444172	−3.511003
C	1.697146	−1.840457	−1.275720
H	4.335913	−1.605337	−1.274884
O	4.962624	−0.206470	−3.423002
C	3.534305	−1.859581	1.311313
C	−0.037871	−0.515583	1.282252
C	−1.001361	−0.508034	2.302151
C	−2.081994	0.579029	0.653163
O	−0.316574	0.384584	−0.973036
O	−0.880587	−0.951894	3.507228
C	1.173362	2.120588	1.823974
C	−1.265044	2.772635	1.720726
C	−2.204751	2.786596	−0.629176

C	−3.693033	2.329702	1.282666
C	−4.589757	0.146532	0.414789
C	−5.076852	−1.025825	1.293403
H	5.721491	0.596044	−1.184682
H	5.303092	1.534682	1.001980
H	2.625469	−1.101816	−4.531012
H	2.229221	−2.786784	−1.115883
H	3.807702	−3.226634	−2.652466
H	4.587486	−2.598744	−4.109578
H	−0.071990	−2.711056	−2.978506
H	4.498901	−1.813103	0.805816
H	0.094087	−1.261776	3.549185
H	−2.957884	−1.172475	−0.189347
H	2.433893	2.234842	0.117487
H	0.331531	3.174889	0.239878
H	−1.588853	3.815716	1.838684
H	−1.255279	2.601946	−1.134733
H	−3.864129	3.288546	1.773680
H	−6.142328	2.894263	1.917785
H	−6.338219	1.256490	2.553936
H	−6.032211	−0.749527	1.751485
H	−5.994464	−2.174091	−0.305060

C	−4.714940	1.480821	1.134440
C	−6.084489	1.833650	1.657514
C	−5.283535	−2.323622	0.515721
H	3.532826	0.523239	2.397973
H	3.132288	0.622443	−2.938194
H	0.948354	−0.667932	−2.898276
H	0.706718	−1.942932	−0.817550
H	3.057212	−3.477479	−4.231171
H	4.837256	−0.014227	−4.366424
H	3.068909	−2.824437	1.084137
H	3.717337	−1.820720	2.385965
H	−3.093032	0.265730	−1.184575
H	3.202828	2.748388	1.605874
H	1.030825	1.666621	2.808126
H	−1.331710	2.305737	2.711026
H	−3.014094	2.448215	−1.281310
H	−2.320806	3.867898	−0.494541
H	−5.283593	0.195608	−0.438423
H	−6.857140	1.618341	0.909306
H	−4.367923	−1.183707	2.114935
H	−5.687249	−3.103498	1.169287
H	−4.353556	−2.708892	0.085761
